# Psychedelic replications in virtual reality and their potential as a therapeutic instrument: an open-label feasibility study

**DOI:** 10.3389/fpsyt.2023.1088896

**Published:** 2023-03-01

**Authors:** Karl Kristjan Kaup, Madis Vasser, Kadi Tulver, Mari Munk, Juhan Pikamäe, Jaan Aru

**Affiliations:** ^1^Institute of Computer Science, University of Tartu, Tartu, Estonia; ^2^Psychiatry Clinic of North Estonia Medical Centre, Tallinn, Estonia; ^3^Institute of Molecular and Cell Biology, University of Tartu, Tartu, Estonia

**Keywords:** psychedelics, virtual reality, therapy, therapeutic mechanisms of psychedelics, altered states of consciousness (ASC), VR-augmented therapy, depression, depressive disorder

## Abstract

**Background:**

Recent research has shown promising results for the therapeutic benefits of psychedelics. One popular view claims that these benefits are mediated by the subjective experiences induced by these substances. Based on this, we designed a virtual reality experience, Psyrreal, that mimics the phenomenological components of psychedelic experiences.

**Aims:**

We aimed to investigate the therapeutic efficacy of Psyrreal and psychedelic VR experiences in treating depressive symptoms as well as explore the effect of Psyrreal on subjective factors which have been suggested to mediate the therapeutic benefits of psychedelics.

**Methods:**

In this open-label feasibility study, thirteen participants with mild-to-moderate depression underwent a 2-day therapeutic intervention implementing Psyrreal. Depressive symptoms were evaluated by the Emotional State Questionnaire (EST-Q2) at the start of the intervention and 2 weeks after. A thematic analysis of semi-structured interviews after Psyrreal was also conducted as an additional assessment of the method.

**Results:**

A 2-day intervention implementing Psyrreal led to significant decreases in depressive symptoms at the 2-week follow-up (*n* = 10, *p* = 0.007, Hedges’ *g* = 1.046) measured by the Emotional State Questionnaire (EST-Q2). The analysis of semi-structured interviews suggests that Psyrreal could lead to insight and alterations in the sense of self in some people.

**Conclusion:**

This work proposes a novel method using virtual reality to augment the treatment of psychological disorders as well as to precisely investigate the mediating subjective factors of the therapeutic effects of psychedelic substances. Our preliminary results suggest that VR experiences combined with psychological support show potential in treating depressive symptoms and further research into similar methods is warranted.

## Introduction

*“I had profound and visionary encounters with nature, and this was long before I conducted my initial experiments with LSD [lysergic acid diethylamide]. Indeed, my first experiences with LSD were very reminiscent of these early mystical encounters I had had as a child in nature. So, you see that it is even possible to have these experiences without drugs.”* Albert Hoffman, cited in Grob (1).

The last two decades have seen a massive resurgence of research into psychedelics largely due to their wide range of therapeutic benefits [e.g., addiction (2–5), depression (6–10), end-of-life distress (11–15), suicidality (16–18), obsessive-compulsive disorder (19), migraine (20, 21), phantom-limb pain (22), for reviews see (23–28)]. This has elevated interest in substance-assisted therapies, where psychedelic sessions are included as a part of the therapeutic process. Preliminary results regarding the efficacy of such methods have been promising (24, 25, 27, 29) and have even shown strong effects in patients whose ailment has not responded to conventional methods (11, 12, 15) [see also (6–10)]. However, it is currently unclear how psychedelic substances confer these benefits. One possibility is that the underlying mechanisms are strictly neurochemical (30, 31). The alternative proposal is that subjective experiences hold the key to the success of psychedelic interventions (32, 33). According to the latter view, therapeutic success is caused by the subjective experiences elicited by these substances, such as mystical (8, 34, 35), ego-dissolution (32, 36–38) or insight experiences (5, 32, 34, 39–41). If psychedelic-induced subjective experiences underlie their therapeutic effects, then it should be possible to achieve at least some of the benefits of psychedelic therapy without the substances themselves (42) simply by emulating specific aspects of psychedelic experiences. Furthermore, despite the many benefits of psychedelic therapy, there can also be some disadvantages to administering psychedelic substances (e.g., cost, legality, and contraindication) in certain population groups (43–45) which highlights a necessity for finding more accessible alternatives. We propose a way to study this question by mimicking many of the wildly different subjective psychedelic experiences in virtual reality (VR), replicating the audiovisual phenomena reported during psychedelic, mystical and deep meditative experiences.

The potential of virtual reality as a therapeutic device has become an increasingly researched topic. Different VR interventions have been used in the treatment of anxiety with promising results (46–49), while depression has only been investigated in a couple of studies (46–49). Recent developments in VR technology suggest that it could be possible to induce mystical experiences (50, 51) and imitate parts of the phenomenology of the psychedelic state (52). Also, some recent studies have suggested that using psychedelic phenomenology in VR can lead to similar cognitive (53) and neural (54) effects as seen under psychedelic substances. Thus, these recent works raise the intriguing possibility that perhaps the implementation of such phenomenology in VR could be used to confer similar therapeutic benefits to psychedelic-augmented therapy.

In this work, we have created Psyrreal, a psychedelic-inspired virtual reality experience. While previous studies (50, 52, 55, 56) implementing psychedelic phenomenology in VR used visually relatively simple and unvarying environments, here we have incorporated a much larger set of visual effects and different environments as psychedelic experiences have massive inter-, and intraindividual variance (57, 58). The participant is taken on an immersive journey through many surreal and vastly different virtual environments which aim to convey certain concepts and narratives often reported during psychedelic experiences [e.g., connectedness (59) or ego-dissolution (60)]. Following the example of Carhart-Harris et al. (12), we conducted an open label feasibility study on healthy adults with mild to moderate depressive symptoms to guide further research into similar methods, and investigate the potential therapeutic effects of Psyrreal as well as the specific mechanisms of VR and psychedelic experiences that may confer these benefits. The primary expected outcome of the study was that a VR experience that simulates psychedelic phenomenology could, in combination with an open therapeutic setting, lead to a decrease in depressive symptoms, measured by the Emotional State Questionnaire (61) (EST-Q2). Expected secondary outcomes included increased reported intensity of mystical experiences [measured by the Revised Mystical Experience Questionnaire (29, 62, 63), MEQ30], psychological insight [Psychological Insight Questionnaire (40), PIQ] and ego-dissolution [Ego-Dissolution Inventory (60), EDI].

## Materials and methods

### Hardware and software

The current version of Psyrreal is a stable release of the virtual reality experience which could already be applied in a therapeutic setting. The experience will run on most modern VR headsets, and HTC Vive Pro Eye was used for development and the experiments. Epic Games Unreal Engine (UE) 4.27 was used as the development platform and the experience was written fully in Blueprint visual scripting language. The final software is distributed as open source upon request^1^, with the Creative Commons Attribution-Non-Commercial license.

**FIGURE 1:**
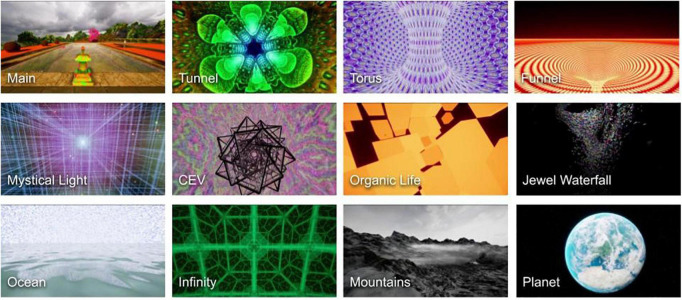
Screenshots of some of the environments in Psyrreal shown in sequence to exemplify the diversity of visuals presented to the user during the experience. The experience starts in a temple **(top left)**, progresses through different levels of varying abstraction and intensity, and culminates in outer space **(bottom right)**. See also Supplementary material 1 for more illustrations of the environments and Table 1 for more detailed descriptions of the implemented concepts. CEV stands for “closed-eye visuals.”

Psyrreal imitates the audiovisual and narrative phenomenology reported during psychedelic experiences (57, 58, 64–74) (Figure 1). Certain elements were also included based on descriptions of pharmacologically induced and spontaneously occurring mystical experiences (29, 72, 75–81), deep meditative states (82–85), and awe-inducing experiences (86–89) as in some cases these offered more details about specific phenomena which also occur in psychedelic states. The phenomenological elements and concepts used in the experience (Table 1 and Supplementary material 7) were selected after careful study of the available literature on psychedelic (57, 58, 64–74), meditative (82–85), awe-inducing (86–89), and mystical (29, 72, 75–81) experiences, written reports (58, 59, 68, 76, 90–92) and visual replications (66, 68, 93) of such experiences.

**TABLE 1:** Overview of elements and concepts common to psychedelic experiences and examples of their implementation in Psyrreal.

Elements of psychedelic experiences	Implementation in Psyrreal
Visual acuity and color enhancement. One of the first alterations often noticed in psychedelic experiences is an enhancement of visual acuity where the visual field appears clearer and sharper and objects become more well-defined (58, 59, 69, 147). This effect is usually accompanied by an intensification and enhanced saturation of colors (58, 67, 69, 92, 148).	The experience starts in a serene, realistic environment overlooking some mountains, with birds singing and calm water flowing around the user (Figure 1 “Main”). After a brief period various open eye visual effects start to appear. Colors become brighter and more saturated; elements of drifting and morphing affect different objects as well as the whole perspective; after-images appear behind falling leaves, and flying butterflies. Certain effects on objects, such as increased contrast, edge aura, texture change and drifting, are gaze-activated (Supplementary Figure 7). Moving the gaze away from the object decreases the effect strength. As the experience progresses, the affected area can change from object edges to whole objects and to the entire scene (e.g., Figure 1 “Jewel Waterfall”).
After-images. Another visual distortion that is often noticed during the early phases of psychedelic experiences is illusory palinopsia, also called after-images, visual tracers, trails or “ghosting” where moving objects leave behind visual trails (67, 149–151).	
Drifting. Objects, parts or the whole visual field often move and distort in many irregular ways, such as drifting, morphing, melting and breathing (69, 92, 147, 152). These effects can start from a slight oscillation of the outlines of specific objects to seamless drifting of textures and objects changing color or morphing from one to another (153).	
Closed eye visuals (CEVs). While it is common to experience geometric patterns on real stimuli with eyes open, intricate patterns are also often reported with eyes closed. CEVs usually start out with simple geometric forms like lattices, cobwebs, honeycombs and spirals (65). Frequently, CEVs include experiences of infinite, kaleidoscopic tunnels formed of geometric patterns and texture repetition (65, 66, 69, 154).	After about 7 min of gradually increasing distortion of the environment, the experience transitions into scenes of CEVs. These start by rather dim 2D patterns which slowly become more vivid and develop into 3D spaces formed of elaborate geometric patterns akin to reports of “DMT hyperspaces” (e.g., Figure 1 “Torus”). Many of these environments also stretch infinitely in all directions (e.g., Figure 1 “Infinity”). The 2D patterns occur recurrently during the rest of the experience, increasing in complexity and acquiring rotating sculptures that generate different mandala-like visuals (Figure 1 “CEV”). One specific example of such patterned scenes are kaleidoscopic rotating tunnels (Figure 1 “Tunnel”), which tend to cause feelings of illusory self-motion and can lead to mild nausea or cyber-sickness (which might, unintuitively, benefit the therapeutic process, see Discussion).
DMT hyperspace. A special kind of visionary experience is the so-called “DMT hyperspace” where people are transported into another world (92, 154). These are often high dimensional spaces (59, 148, 154) which contain massive or even infinitely large cathedrals, machines capes or abstract spaces made of geometric patterns (154). The passage into this space, a “DMT breakthrough,” is often accompanied by sensations of overwhelming intensity, fast or accelerating movement along geometric tunnels and an ascending or intensifying sound (74, 92, 154–156).	
Visions. With higher doses and during more acute phases of psychedelic experiences, CEVs can become increasingly lucid, hyperdimensional with more complex patterns, and acquire profound meaning (69, 92). Often dreamlike (70, 73) visions arise which can include whole scenes or landscapes, autobiographical memories or imagined realistic situations, as well as mythical or archetypal imagery (57, 58). Such visions are sometimes experienced in a synesthetic fashion, i.e., not just seen or imagined visually, but also “felt” (57, 58, 91).	While many levels consist of abstract shapes and patterns (e.g., Figure 1 “Organic life”), we also included levels that simulate elements of more coherent visions. “Visions” induce the feeling of being in a completely different environment from the main level. We included views of landscapes and grandiose and vast scenes (e.g., Figure 1 “Ocean”), involving large cathedrals and a mountain ridge with a floating monastery (Figure 1 “Mountains”). We also implemented the “Overview” (157–159) and “Ultraview” effects (160) where, respectively, the subject experiences an overview of the Earth (Figure 1 “Planet”) and thereafter the whole Universe.
Mystical experiences. Moderate and higher doses of psychedelics often result in participants having mystical experiences (29, 72, 77, 79). These indescribable and paradoxical experiences are often described to contain a felt union with God, Nature or the Universe, receiving transformative insights and feelings of profound peace and bliss (72, 80, 81). At the core of mystical experiences is losing a sense of individual self (see also ego-dissolution) and “becoming one” with objects of attention or with “everything” (72, 80). Another common mention is that of a bright white or golden light that could be seen or “felt” in a synesthetic nature (72, 82, 161). Mystical experiences also often contain alterations and transcendence of time and space, and feelings of vastness, awe and sacredness (58, 62, 72, 80, 92).	The synesthetic and phenomenologically barren nature of mystical experiences creates a significant hurdle in representing them in the audiovisual medium of VR. Nevertheless, we implemented a recurrently appearing level with a bright white light and calm and sacred music (Figure 1 “Mystical Light”). We also included a level with spherical particles that circle around the player, giving the impression that the environment is “alive” and interacting with the player. Psyrreal also has a soundtrack with varying tempo and intensity to induce a sense of temporal alteration (see also “Overview of Psyrreal VR”).
Ego-dissolution. Psychedelic experiences also bring about alterations of the sense of self which are often discussed under the term ego-dissolution (or “ego-death”) (57, 162–166). This is often reported as a dissolution of the embodied self, disintegration of self-related thoughts and felt ownership of thoughts (60, 164, 167), and/or cessation of implicit subject-object distinction as the subject feels as “one” with their surroundings (57, 72).	To emulate ego-dissolution, we included a virtual body representation of the user in certain levels which consists of a sphere that mimics the environment, thus creating a sense of connectedness to the virtual “world.” At the culmination of the experience the particles of the universe converge at the position of the subject to then explode outward and fade, disintegrating the virtual self.

The participants begin the experience in a virtual “real world” which starts to acquire psychedelic phenomenology and continues to progress through a total of 19 distinct visual levels incorporating and combining various psychedelic effects. The visuals for the most part consist of abstract shapes and geometric patterns that would allow participants to project their own meaning to the experience. The duration of levels varies from approximately 30 s to 2 min. A specific soundtrack was composed for Psyrreal implementing narrative (e.g., varying intensity) and perceptual (e.g., temporal alterations) aspects of psychedelic phenomenology matching the visual levels. Psyrreal was validated among participants with extensive psychedelic experiences who evaluated its similarity to psychedelic experiences (see Supplementary material 3 for more details). All participants had prior experiences with lysergic acid diethylamide (LSD) and psilocybin with five (out of seven) participants also having prior experiences with *N,N*-dimethyltryptamine (DMT) or Ayahuasca. Participants in the validation study were recruited *via* social media. Five people rated at least one scene from Psyrreal to be visually very similar (4 on a scale from 0 to 5) to psychedelic experiences. Though the experience is purely audiovisual and did not include any physical stimulation, six people also rated the physical sensations induced by Psyrreal to be very similar to those experienced under the effects of psychedelics.

### Therapeutic intervention using Psyrreal

#### Participants

The sample consisted of 13 participants (8 women, 5 men) of ages 20–58 (*M* = 33.8, *SD* = 10.6; further information on the participants can be found in the data files, linked under Supplementary material). The study was advertised on an Estonian website that promotes discussion about mental health (peaasi.ee) and recruitment was conducted through self-referral from 20th October 2021 until 8th December 2021. The sample size was determined by practical constraints and concurrent COVID-related restrictions as well as sample sizes of similar feasibility studies [e.g., (12)]. Applicability was decided based on their scores on a self-rated depression screening questionnaire (depressive scale cut-off of ≥12, see below) administered during online registration. The scores of the questionnaire were analyzed and updated with a clinical psychologist on the first day of experiments. Additional criteria for exclusion were: hypersensitivity to motion sickness, a diagnosis of psychotic disorders and schizophrenia, a history of epileptic seizures or psychotic episodes, or a family history of schizophrenia. We also excluded people who were currently undergoing treatment for depression (therapy or medication). Most participants had a previous diagnosis of depression (9 out of 13), but participants who were currently receiving treatment were excluded (see also additional analyses Supplementary material 2). All participants had normal or corrected to normal vision. Participants were asked to refrain from consuming alcohol or other substances before the experience to avoid cybersickness and potential confounding.

The study was approved by the ethics committee of the University of Tartu and performed in accordance with relevant guidelines and regulations. Participants gave written informed consent prior to participation. All 13 registered people finished the study. One participant (P6) was excluded from the analyses due to receiving antidepressant therapy at the time of the study and another was excluded due to their updated score of depressive symptoms being below the cut-off, thus leaving the final sample at 11 participants. Note that an estimated effect size of 1.11 (based on a relatively similar approach using VR to treat depression) should be detectable with a sample size of 9 [for a statistical power of 0.8, found with G*Power 3.1 (94)].

#### Measures

The Emotional State Questionnaire (61) (EST-Q2), a standard self-report tool for depression screening in Estonia (95), was used to evaluate differences in symptoms before the experiment and at a 2-week follow-up. A cut-off of ≥12 (threshold commonly used in clinical practice) was applied to only include participants with symptoms indicative of clinical depression. We also evaluated the response (proportion of participants showing decrease of at least 50% of baseline) and remission rate (proportion of participants showing decrease to below 12) based on EST-Q2 scores. The Montgomery−Åsberg Depression Scale (96) (MADRS) was administered by the psychologist before the experiment to validate the depressive symptoms of our sample.

**FIGURE 2:**
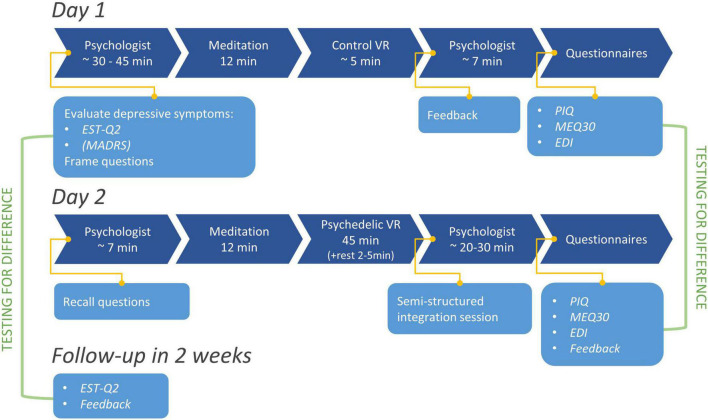
An illustration of the procedure of the experiment on day 1 and day 2, as well as measurements conducted on the 2 days of experiments and during the follow-up after 2 weeks. The green lines highlight the comparison groups used for the statistical analyses.

One important facet of subjective experiences that could be instrumental for therapeutic benefits is the insight experience (5, 32, 40, 41). The Psychological Insight Questionnaire (40) (PIQ) was used to capture insights in our sample at the end of the first (control) and second days (Figure 2; same for the other following questionnaires). Another aspect of subjective experience that has been suggested to underlie the beneficial effects of psychedelic therapy is ego dissolution (32, 38, 97), measured here by the Ego-Dissolution Inventory (60) (EDI). Mystical experiences have also been proposed as an important mediating factor for the benefits of psychedelic compounds. Here we used The Revised Mystical Experience Questionnaire (29, 62, 63) (MEQ30) to measure the intensity of mystical experience.

MEQ30, PIQ, and EDI were administered at the end of the first day and again at the end of the second day. EST-Q2 was administered during registration and 2 weeks after the experiments. More detailed descriptions of the questionnaires can be found in Supplementary material 5. Baseline scores were also reevaluated during the first discussion with the clinical psychologist at the start of the first day. A semi-structured interview about the experience was also conducted by the clinical psychologist at the end of the second day. An additional background and feedback questionnaire was administered at the end of the second day of experiments, and a feedback form was sent to the participants 2 weeks after the experiments. All questionnaires were administered in Estonian.

#### Study procedure

This open-label feasibility study used a one-group, uncontrolled, longitudinal design to investigate the effects of psychedelic phenomenology in VR. Experiments were conducted on two consecutive days (Figure 2; see also Supplementary material 6 for more details on the experimental setting). The first day included a diagnostic and preparative 30–45 min session with a clinical psychologist, discussing the depressive symptomology of the participant based on their scores on the EST-Q2 Depression scale (filled in during the online registration) and guiding the participants to frame questions and think about their worries. Afterward, the participants were instructed on how to use the VR equipment and partook in a demonstrative 15 min VR experience, which consisted of a 10 min guided meditation (in a cave-like environment, Supplementary Figure 10; however the participants were instructed to close their eyes for the duration of the meditation) and a further 5 min of being in a non-interactive living-room environment (default Steam VR Home room, Supplementary Figure 9). Participants were seated comfortably during both VR experiences on day 1 and 2. Then the participants had another shorter (5–10 min) discussion with a clinical psychologist and filled in the rest of the questionnaires (EDI, PIQ, and MEQ30). The demonstrative VR experience on the first day also served as a control condition for comparisons with EDI, PIQ, and MEQ30 measures. Participants were instructed to answer the questionnaires based on the 5 min spent in the living-room VR environment. EST-Q2 was not administered after the control VR as it is not a suitable measure for short term changes in mood.

The second day had a similar structure starting with a short discussion with the psychologist to evaluate the effects of the first day and to prepare the participant for the VR experience. During this, the participant was also instructed to look around freely during the experience, sit however they felt comfortable, and not to overly focus on their questions but rather to relax and focus on the experience itself. Then, the participant underwent the same guided meditation in VR as the day before which was directly followed by the 45 min long VR experience. The psychologist was waiting in the next room in case of any psychological emergencies, and a technician was present in the room with the participant to monitor the VR system. After the experience, the participant was allowed to rest for as long as they preferred and then had an integrative session (20–30 min) with the psychologist and answered the questionnaires (EDI, PIQ, and MEQ30). Follow-up EST-Q2 questionnaire was sent to the participants 2 weeks after their participation in the experiment.

The experiments were conducted in the virtual reality laboratory at the Institute of Computer Science of the University of Tartu between 29th October 2021 and 11th December 2021. For more additional details on setting and study procedure see also Supplementary material 6.

### Statistical analysis

Paired sample *t*-tests were conducted on the before and after scores of the questionnaires (EST-Q2, EDI, PIQ, and MEQ30). All tests were two-tailed with α = 0.05. Shapiro–Wilk test confirmed approximately normal distribution for the before-after differences of all datasets. All statistical analyses were conducted with JASP (98) (version 0.16.1.0).

One participant did not fill in the questionnaire at the 2-week follow-up and was therefore excluded from the analyses concerning the EST-Q2 measure. Additionally, two participants implied in their follow-up report that they had sought psychological counseling after the intervention. Because of this, we conducted an additional analysis on all questionnaires, excluding the two participants, yielding similar results which are reported in the Supplementary material 2.

## Results

### Therapeutic intervention

We conducted an open-label feasibility study in adults (*n* = 11) with mild to moderate depressive symptoms. Note that *n* = 10 for the EST-Q2 measure, as one participant did not complete the follow up questionnaire. The experiments took place on two consecutive days with Psyrreal being implemented on the second day (see Figure 2). No adverse effects were reported during the administration of the VR experience, even though the participants were encouraged to express any discomfort. In the interviews afterward, four participants (out of 13) mentioned transient nausea during the VR experience.

**FIGURE 3:**
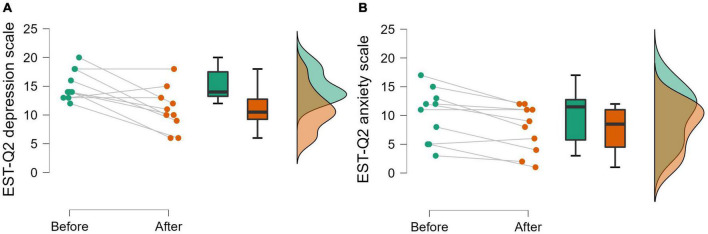
A raincloud plot of the EST-Q2 depression scale **(A)** and anxiety scale **(B)** scores as measured before the experiment and 2 weeks after. Depicted are individual scores, as well as boxplots and density distributions of the results (*N* = 10).

Baseline EST-Q2 depression subscale results were *M* = 15.20 (*SD* = 2.66) and showed good consistency with the MADRS questionnaire results (*r* = 0.66, *p* = 0.010). There was a significant decrease in EST-Q2 scores at the 2 week follow-up (*M* = 11.00, *SD* = 3.74; paired samples *t*-test, *t*_(9)_ = 3.50, *p* = 0.007, Hedges’ *g* = 1.046; Figure 3A). The response rate of the treatment was 18% and remission rate was 60%. The difference between the response and remission rate is largely due to the mild-to-moderate symptoms reported by the studied population. The anxiety scale results of the EST-Q2 showed a similar pattern, as scores decreased from baseline *M* = 10.10 (*SD* = 4.65) to *M* = 7.60 (*SD* = 4.14) after 2 weeks [*t*_(9)_ = 3.73, *p* = 0.005, *g* = 0.459; Figure 3B].

PIQ score results were *M* = 24.74 (*SD* = 22.98) after the control condition and *M* = 29.09 (*SD* = 21.73) after the experimental condition. Paired samples *t*-test indicated that the change was not statistically significant [*t*_(10)_ = 1.256, *p* = 0.238, *g* = 0.161].

EDI scores increased from *M* = 35.76 (*SD* = 21.14) after the control condition to *M* = 39.83 (*SD* = 27.77) post-experiment, but the change was not statistically significant [*t*_(10)_ = 0.829, *p* = 0.426, *g* = 0.136]. Two participants (P2, P3) did show a large increase (>25 points) in EDI scores.

The MEQ30 average score increased from *M* = 39.58 (*SD* = 19.57) to 42.73 (σ = 18.43). The change was not statistically significant [*t*_(10)_ = 0.673, *p* = 0.520, *g* = 0.137]. One participant (P3) reported a complete mystical experience (all MEQ30 subscales over 60) on day two. One participant (P7) reported a complete mystical experience on the first day, but not on the second.

While the changes of scores on PIQ, MEQ30 and EDI did not reach statistical significance, the results were highly varied. A thematic analysis (99) was conducted based on the transcripts of the integrative semi-structured interview sessions to investigate the effects of the intervention on individual participants. Extracts from the transcripts that were used in the thematic analysis can be found in Supplementary material 4. The analysis revealed certain themes that were often mentioned in the interviews (Table 2). All participants reported feeling some positive emotions during the experience with 55% of participants reporting calmness or peace and the same amount of people reporting feelings of joy, pleasure, or fun. Seven participants also reported negative emotions (sadness, fear, and anxiety) with four participants mentioning feeling sadness. A total of 45% of participants reported that they had personally relevant thoughts during the experience, for example, one participant mentioned: “*From this place where I got in touch with this emotion [sadness] these other questions also started to unravel and I understood many of my [behavioural and cognitive] patterns*,” (P12). Six participants mentioned that they did not reach any new understandings, while three did arrive at new beneficial understandings: “*[.] this novel understanding that I can reprogram myself, that this essence of myself doesn’t exist—it’s very liberating. It gives me, in some sense, a vitality that I’m looking for,” (P7).*

**TABLE 2:** Common themes found in the analysis of the semi-structured interviews (*n* = 11). The colored bar plot indicates the proportion of participants who mentioned that theme in their interviews.

Theme	# of participants
Positive emotions	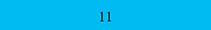
Negative emotions	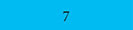
Alterations in sense of self	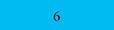
Feelings of joy/pleasure	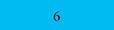
Feelings of calmness/peace	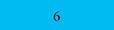
Tiredness / Sleepiness	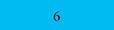
No novel insights	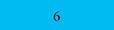
Thought-provoking	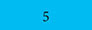
Changes in perspective	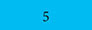
Loss of VR presence	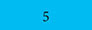
Comparison to games/movies	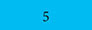
Ambiguous tension	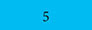
Somatic effects	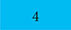
Ineffability	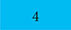
Alterations in embodied sense of self	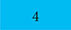
Sadness	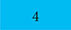
Fear	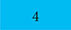
Cybersickness / Nausea	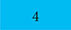
Emotional tension	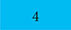
Feeling of insight	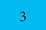
Intellectual insight / Understanding	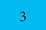
Alterations in narrative sense of self	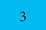
Technical issues	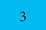
Boredom	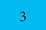
Physical tension	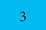

Three participants specifically highlighted a physical feeling of insight as illustrated by the following quote from participant seven: “*It was a different kind of insight, like a new insight. Like a physical insight*,” (P7). Five people reported changes in perspective: “*[.] Like a shift in perspective - in the beginning, I was looking more at the details, afterward I was looking at the whole*,” (P3). Six participants reported alterations in their sense of self with three reporting alterations in the narrative sense of self and four participants reporting alterations in the embodied sense of self. For example: “Did you experience any change or loss of your self-image or sense of self? *Yes, like dissolution, yes. [.] The whole experience was so immersive that you melted into it*,” (P2). A total of 36% of participants reported somatic effects during the experience: *“Specifically the parts with strong motion, these almost physical experiences, those were the most impactful*,” (P12).

## Discussion

We created a novel VR experience based on psychedelic and mystical experiences as well as meditative states and implemented it in a therapeutic intervention for people with mild-to-moderate depression.

The results of the study suggest that using VR experiences in a therapeutic setting could be beneficial in treating depressive symptoms, with participants showing a significant reduction in depressive symptoms 2 weeks after the experiments. As this was an open-label feasibility study with a relatively small number of participants, strong conclusions about the efficacy of such interventions cannot be made. Nevertheless, as 73% of participants showed a decrease in their depressive symptoms 2 weeks after a short 2-day intervention at a 60% remission rate, further research into such methods is warranted. Additionally, a previous study using a VR experience to treat depression also reported a comparable effect (49), offering preliminary tenability for the potential therapeutic benefits of VR experiences. Anxiety scores also decreased significantly at the follow-up. However, the anxiety scores were generally low beforehand with four participants having a score indicative of an anxiety disorder (≥12). That being said, these results suggest using psychedelic-inspired virtual reality for the treatment of anxiety to also be a promising avenue for research. Additionally, results from the thematic analysis suggest that a psychedelic VR experience, when accompanied by a dedicated therapeutic setting, might be able to induce insight and understanding at least for some people. Three participants reported gaining new and beneficial insights related to their problems and two more mentioned gaining new perspectives during the experience. However, across sample the change in insightfulness rating as measured by PIQ did not reach significance, indicating great interindividual variance.

Overall, these results add some tentative support to the hypothesis that subjective experiences mediate the benefits of psychedelics. Psyrreal incorporates a wide range of elements common to psychedelic experiences such as specific audiovisual phenomenology (e.g., visual acuity enhancement and CEVs; see Table 1), different structural elements of these experiences [e.g., overwhelming and oscillating intensity, and progression of the experience (100)], and general experiential themes (e.g., mystical and ego-dissolution experiences; see Table 1). The effects of our intervention on depressive symptoms were similar (albeit weaker) to those seen in psychedelic-assisted therapy (101). While the questionnaires implemented to evaluate the subjective factors which have been found to mediate the therapeutic benefits of psychedelics did not show statistically significant changes, the results of these questionnaires were highly varied in our small sample and prevent us from making strong conclusions. However, the results of the thematic analysis suggest that implementing surreal awe-inducing environments, and intense and variable experiences in VR could facilitate insights or alterations in sense of self at least for some people. Future studies are required to confirm these speculations and evaluate the mediating factors with a larger sample and a randomized placebo-controlled trial design.

Previous similar studies using VR (50, 51) have found slightly higher results on the MEQ30 questionnaire. Specifically, the scores of the Mystical and Positive Mood subscales were higher for Isness-C (for Isness-D only the Positive Mood subscale was significantly higher) (51). However, these studies also implemented a guiding narration by a “trained drama therapist” which emphasized elements of mystical experiences (50) potentially enhancing the perception of the experience as mystical. While the current version of Psyrreal did not include narration, adding it in the future could further enhance the effect of Psyrreal. The differences on the Positive Mood subscale are likely to be influenced by the symptoms of depression prevalent in our sample and the “amplifying” effect of the experience which allowed some participants to get in touch with their negative emotions.

### Possible mechanisms of psychedelic-inspired-VR-augmented therapy

Our augmented therapy intervention combined a therapeutic environment and discussion about one’s problems with an engaging VR experience. One potential mechanism how this intervention yields therapeutic benefits could be that the intense and different virtual experience amplifies the therapeutic process and provides an experiential route for the participant to get in touch with their emotions (102–106) as an alternative to cognitive therapies which emphasize the role of explicit verbal discussion and reasoning (107–109). According to current understandings of psychedelic therapy, psychedelic substances in contrast to conventional antidepressants serve to amplify mental content and thus help to address rather than avoid aversive memories and emotions (23, 59, 110). Some participants mentioned that they were able to access feelings that have otherwise stayed out of reach, for example one participant expressed to the psychologist: “*When we met yesterday, [.] I said I could not feel sadness. We could have five more similar sessions and I still wouldn’t feel sadness. But thanks to this experience, as short as it was yesterday, suddenly this sadness arose (in me)*,*” (P7).*

A related possible mechanism is that the intensity of the experience can require relinquishing control which could result in some degree of relaxation of fixed cognitive constraints. The relaxation of top-down beliefs is suggested to be one of the possible mechanisms behind the therapeutic effects of psychedelics, wherein potentially pathological content can be accessed and overwritten (111). Letting go of control and allowing oneself to experience anything that comes up in a psychedelic experience could allow more difficult content to reach consciousness, shorten the challenging episodes and foster integration and healthy interpretation of difficult content (112, 113). This concept of “letting go” is a central element of psychedelic (59, 113, 114) and other forms of therapy (115, 116), ritual use of psychedelics (117) as well as an important part of meditative practices (84, 118–120) [see also (41)]. Psyrreal mimics the oscillating and overwhelming intensity of psychedelic experiences (68). For example, some sections of Psyrreal purposefully depict high-motion visual scenes without actual physical motion that induce quite strong physical sensations and mild symptoms of cybersickness that can be “let go” (e.g., Figure 1 “Tunnel”). Hence, this somatoaudiovisual intensity could also function as something that could be accepted or surrendered to. The beneficial effects of “letting go” were also remarked by some participants, for example: *“[.] Then, at one moment, I let go, thinking that it doesn’t work like this [focusing on finding answers]. [.] Then it actually started to work, yes. [.] I think that the first half of the experience you kind of get into it or start going along with it and then the answers arrive very clearly somehow*,” (P12). In our study, participants were explicitly instructed to accept and let go of any emotions that might arise during the experience. While Psyrreal does not include any explicitly affective visual stimuli (the music, however, varies in its emotional tone) most participants mentioned experiencing a wide range of emotions with some explicitly reporting “getting in touch” with their emotions (see above). Therefore, we suggest that another potential therapeutic mechanism for this type of therapy could be combining a virtual emotion-eliciting experience with the preparation of “letting go.” Somewhat similar ideas have been implemented in VR-augmented exposure therapy where people are exposed to aversive virtual stimuli to facilitate habituation, inhibition or cognitive reappraisal of the psychological reaction (121–123).

While the experience of being in virtual reality in itself might facilitate elements that could beneficially augment the therapeutic process, there could also be added value in implementing specific visual content from reports of subjective psychedelic and mystical experiences. Unusual, infinitely vast, and even surreal stimuli common to such experiences might result in emotions of surprise and awe, where the novel stimulus needs to be accommodated into existing cognitive structures (86). The necessity for accommodating vastly different and novel stimuli would require updating the existing mental framework (86, 124) and thereby lead to a state of plasticity or “insightfulness” (41). This could allow the subject to gain different perspectives, revise pathological beliefs, and come to new insights (105, 124, 125). Such effects have also been discussed in contemporary psychedelic science (111). In fact, awe has been suggested to be a central mediator of the beneficial effects of psychedelics (126) and a potential therapeutic asset for different mental health disorders, including depression (127). Psyrreal contains infinite abstract “worlds” as well as vast landscapes and cathedrals which could induce awe (87). Additionally, virtual environments which break the usual laws of physics might also require accommodation and induce states of relaxed beliefs (105, 128, 129).

### Limitations

Despite our efforts, we are far from claiming that Psyrreal is an exact replication of psychedelic experiences. Psychedelic experiences encompass many aspects of consciousness (32, 57, 58) that we tried to replicate within the audiovisually confined medium of virtual reality. Some elements of substance-induced psychedelic experiences are inherently impossible to be implemented in virtual reality (e.g., hyperdimensional spaces, many synesthetic elements). Also, compared to psychedelics, virtual reality has some additional aspects which could contribute to the loss of immersion in the experience. For example, people might lose their immersion due to interactive elements that fail to meet their expectations or due to the graphics not being realistic enough (130).

The open-label design, small sample size and lack of controls in our reported experiment do not allow us to make strong claims about such interventions yet. We were unable to reliably estimate the effect of the control condition on EST-Q2 scores due to the 2-day design, as such short term changes (or lack thereof) would be unlikely to reflect true effects. Hence, the main comparison in depression scores could only be evaluated at least 2 weeks after the intervention. The design was chosen after careful consideration of how to best optimize resources for the purpose of evaluating preliminary results and feasibility for conducting more expensive future studies – which we now hope to conduct. Therefore, it is also possible that an expectancy bias in the participants, initial discussion with the psychologist, guided meditation or even a brief experience of non-psychedelic virtual reality might be responsible for the decrease of depressive symptoms. This is highlighted by a few participants reporting quite high results on the Psychological Insight Questionnaire and Mystical Experience Questionnaire even on the first day. While the psychologist did not apply usual therapeutic techniques and assumed the role of an observer, simply an opportunity to discuss their problems could already have had a beneficial effect.

While MEQ30 has been validated for psychedelic compounds and it has seen some use in investigating non-pharmacological methods for inducing mystical experiences [e.g., (50, 51, 131–134)], we are not aware of any studies using non-pharmacological methods that have implemented a control/baseline measurement of MEQ30. Our results suggest some potential difficulties in using the MEQ30 with non-pharmacological methods, as multiple participants had confusing results with one participant reporting a complete mystical experience on day one and a further two participants who only very narrowly missed out on the threshold of a complete mystical experience. While it is not impossible that they actually had a real mystical experience on the first day, it is unlikely and they also did not describe their experience as such during their discussion with the psychologist. We speculate that certain inflated scores could be due to the subjective nature of the questionnaire, and/or difficulties in understanding the questions. As the MEQ30 instructions require people to compare a specific experience (in our case, the VR experience) to other previous experiences in their lives, the results are dependent on the intensity of similar experiences they have had in the past, as well as their previous experience with VR. This also relates to difficulties in understanding the questions: people with varying amounts of mystical experiences can have a very different understanding of the questionnaire items which is further exacerbated by the fact that mystical experiences are ineffable and hard to describe (35, 81). It is also possible that a VR experience by itself could incline some participants to report high scores on the MEQ30. Finally, it is possible that the participants scored higher due to not wanting to disappoint the experimenters, as the people conducting the experiment were friendly and supportive to create an environment where participants feel safe and comfortable. Though, due to the open-label design, participants were aware that the measurements on the first day served an introductory and demonstrative purpose.

The guided meditation and short demonstrative VR (used on the first day) could also be conducive to reporting high scores on the MEQ30, PIQ, and EDI. Additionally, the results of these questionnaires were compared between a 5 min long control VR and 45 min long Psyrreal, which limits the comparability of the conditions. The effects of meditation on inducing altered states of consciousness, insight, self-alterations and mystical experiences are well documented (82–84, 132), but even a short experience with VR could have some effect for a couple of reasons. First, for people who have not had much experience with VR, the experience might be something so different that it has a strong effect on them (135). Second, the content of VR might by chance be specifically relevant to the participant, as was the case for one participant (who was excluded from the analysis) who had been pondering whether they should go traveling to the mountains with their ex-spouse. As the demonstrative VR was in a small room with a balcony view onto a mountain range the participant felt it to be very personally meaningful. Third, one of the potential mediators of the beneficial effects of psychedelic experiences as well as a crucial element of mystical experiences (32, 38, 97) — ego-dissolution, might be induced to some degree in any VR experience with an altered image of the body (136, 137). While the EDI did not show a significant change from baseline, half of the participants showed relatively high (a score of over 40) results even after the demo experience on the first day and several participants mentioned alterations in their sense of self during the integrative sessions. Therefore, it can be difficult to disentangle the effects of VR and the specific psychedelic content of our designed experience. Also, blinding procedures for such methods are complicated by a bane common to psychedelic investigation — the active condition is easily distinguishable from the control condition (44, 138, 139). The issues of blinding and confounding are important to be addressed in future studies.

### Advantages of virtual reality for studying psychedelic therapy

Although virtual reality is likely a less powerful tool than psychedelic substances (52) [but see (50)], using it to augment therapy could be beneficial for those who suffer from acute side effects of psychedelics or who prefer to avoid consuming these kinds of substances. Virtual reality could also work as a stepping stone before engaging in psychedelic therapy (101) and could be used in countries where psychedelics are not available for medical use. The duration of virtual reality experiences is also less constrained than psychedelic experiences, which (in the case of LSD) can last over 16 h (140). Virtual reality experiences can be stopped at any moment (e.g., in the case of challenging experiences) or paused if the participant would like to immediately discuss something. Virtual reality could also be a powerful tool for investigating the precise therapeutic factors of subjective (e.g., psychedelic) experiences (101) which are often confounded and contain multiple interfering interactions, as it allows to test and separate different elements and concepts that might mediate beneficial effects. In other words, virtual reality allows one to study in a controlled manner which specific subjective experiences (e.g., certain visual experiences, challenging experiences, ego-dissolution) might be beneficial for therapeutic success.

Forms of meditation, audiovisual media, engaging in physical activities (e.g., dancing and hiking) could also be used as therapeutic “amplifiers” (105), but virtual reality offers an especially powerful medium for such a tool. First, it can offer a strong sense of presence and immersion in a specific environment which might be beneficial for the efficacy of such augmentation (101). Second, virtual reality is adaptable and could be tailored to the needs and specific symptomatology of the participant as well as to different disorders (e.g., anxiety and post-traumatic stress disorder). For instance, in Psyrreal it is possible to remove and add specific parts of the experience to customize the virtual reality experience for the specific participant and the circumstances. Third, virtual reality could have some advantages in regard to inducing ego-dissolution (51, 105, 136, 141, 142) which could be one of the mechanisms behind the therapeutic effects of psychedelics (32, 38, 97). Fourth, virtual reality assisted therapeutic interventions have high satisfaction rates (143) and are often preferred by patients. This was also echoed by our participants who subjectively evaluated the potential effectiveness of such an intervention with a mean rating of 8.00 out of 10 (*n* = 12, *SD* = 2.30).

### Future developments

The current study highlights multiple important directions for further research. First, some participants in our study mentioned that the computer graphics and lack of interactivity led to a loss of immersion in the experience. Next iterations of Psyrreal might benefit from adding a guided narration to make certain implemented concepts (e.g., connectedness or ego-dissolution) clearer, and from incorporating more interactive elements which could be beneficial for increasing immersion in the experience (144). Technical developments in the growing field of virtual reality are likely to hugely enhance designing such therapeutic experiences and could be used to develop more immersive and ‘realistic’ experiences in the near future. Second, while Psyrreal implements a wide variety of different phenomenological aspects, it could also be interesting to investigate specific components separately to help elucidate the therapeutically beneficial factors of psychedelic experiences. Furthermore, implementing an appropriate control condition is a difficult task for VR methods which could be addressed in future research (i.e., developing suitable “placebo” VR experiences). Third, combining virtual or augmented reality with, for example, sensory deprivation methods or microdosing of psychedelics might also offer intriguing avenues of research (101, 145, 146). Fourth, the results of MEQ30 and EDI highlight some issues in using these questionnaires for VR experiences (see “Limitations”). The development of new questionnaires or adaptation of existing ones for VR could be useful to investigate the effects of similar VR-based methods more reliably.

## Conclusion

We developed Psyrreal, a somatoaudiovisual virtual reality experience based on psychedelic and mystical phenomenology to aid the treatment of different psychological disorders and help participants to see things from new perspectives. We observed that using this novel therapeutic tool in an augmented therapy intervention alleviates mild-to-moderate depressive symptoms. The results of the study suggest that psychedelic subjective experiences implemented through virtual reality could have therapeutically beneficial effects (potentially extending beyond depression) and that further research into similar novel tools is warranted. Implementing elements of psychedelic experiences that potentially mediate therapeutic effects in virtual reality can also help to precisely investigate and elucidate the mechanisms underlying psychedelic therapy.

## Data availability statement

The original contributions presented in this study are included in this article/Supplementary material, further inquiries can be directed to the corresponding authors.

## Ethics statement

The studies involving human participants were reviewed and approved by Ethics Committee of the University of Tartu. The participants provided their written informed consent to participate in this study.

## Author contributions

KK, MV, and JA designed the software. KK, MV, JP, and KT conducted the experiments. JA conceived the idea. All authors contributed to the writing of the manuscript, designing the experiments, and approved the submitted version.

## Footnotes

1 Can be requested at the website https://psyrreal.mozellosite.com.
