# Corrigendum: Psychedelic replications in virtual reality and their potential as a therapeutic instrument: an open-label feasibility study

**DOI:** 10.3389/fpsyt.2023.1198103

**Published:** 2023-05-09

**Authors:** Karl Kristjan Kaup, Madis Vasser, Kadi Tulver, Mari Munk, Juhan Pikamäe, Jaan Aru

**Affiliations:** ^1^Institute of Computer Science, University of Tartu, Tartu, Estonia; ^2^Psychiatry Clinic of North Estonia Medical Centre, Tallinn, Estonia; ^3^Institute of Molecular and Cell Biology, University of Tartu, Tartu, Estonia

**Keywords:** psychedelics, virtual reality, therapy, therapeutic mechanisms of psychedelics, altered states of consciousness (ASC), VR-augmented therapy, depression, depressive disorder

In the published article, there was an error in the author list, and author Mari Munk was erroneously excluded. The corrected author list appears below.

Karl Kristjan Kaup^1*^, Madis Vasser^1^, Kadi Tulver^1^, Mari Munk^2^, Juhan Pikamäe^1, 3^ and Jaan Aru^1*^

^1^Institute of Computer Science, University of Tartu, Tartu, Estonia

^2^Psychiatry Clinic of North Estonia Medical Centre, Tallinn, Estonia

^3^Institute of Molecular and Cell Biology, University of Tartu, Tartu, Estonia

The authors apologize for this error and state that this does not change the scientific conclusions of the article in any way. The original article has been updated.

