# Supplementary material for: Psychedelic replications in virtual reality and their potential as a therapeutic instrument: an open-label feasibility study
Source: Front Psychiatry. 2023 Mar 1;14:1088896. doi: 10.3389/fpsyt.2023.1088896 (PMC10022432; doi:10.3389/fpsyt.2023.1088896)
Supplement: Supplementary file 1 [file Data_Sheet_1.PDF]

Supplementary materials for “Psychedelic replications in virtual reality and their therapeutic effects on depressive symptoms”

K. K. Kaup[1], M. Vasser[1], K. Tulver[1], J. Pikamäe[1,2], J. Aru[1]

[1] Institute of Computer Science, University of Tartu, Estonia
[2] Institute of Molecular and Cell Biology, University of Tartu, Estonia

Tartu, 2022

Table of Contents

SM1. Additional figures of environments used in Psyrreal	2
SM2. Additional EST-Q2 analyses	7
SM3. Validation of Psyrreal	9
SM4. Thematic Analysis	10
SM5. Measures	23
SM6. Experimental Design	24
SM7. Overview of Psyrreal VR	25
References	26

SM1. Additional figures of environments used in Psyrreal

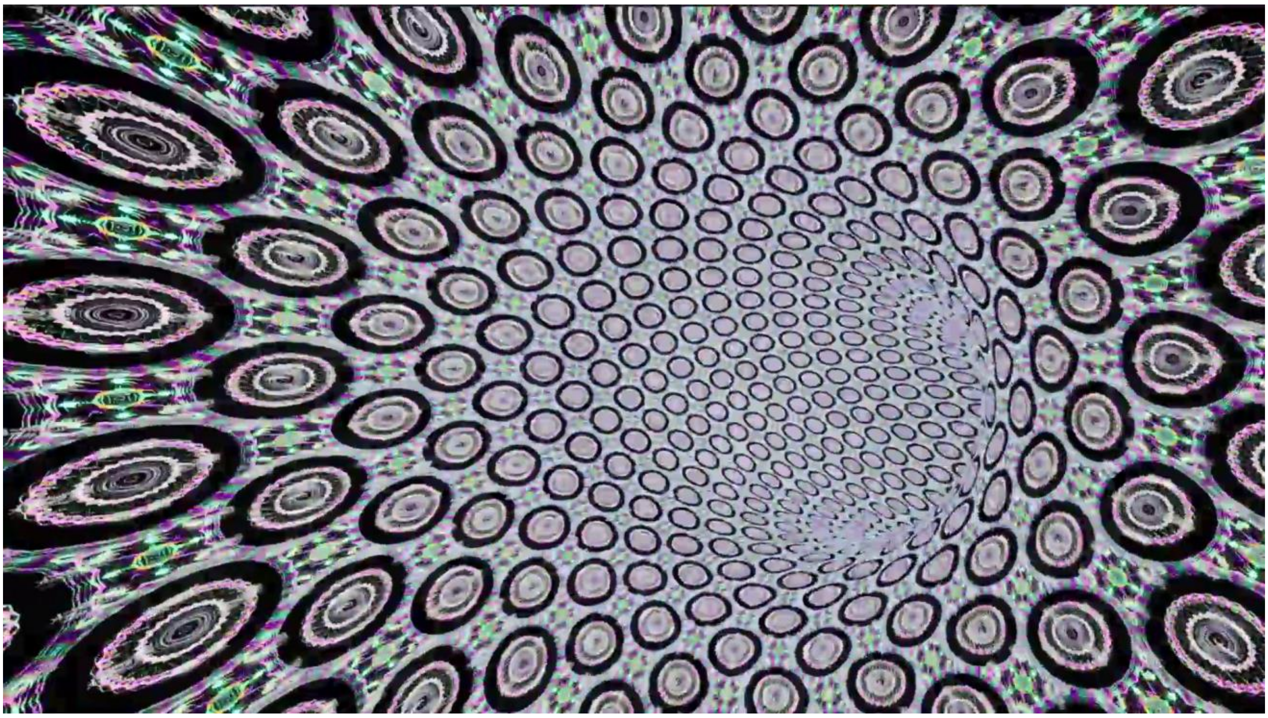

Figure S1. Screenshot of the Torus level. The environment mimics DMT hyperspaces in a patterned toroidal structure.

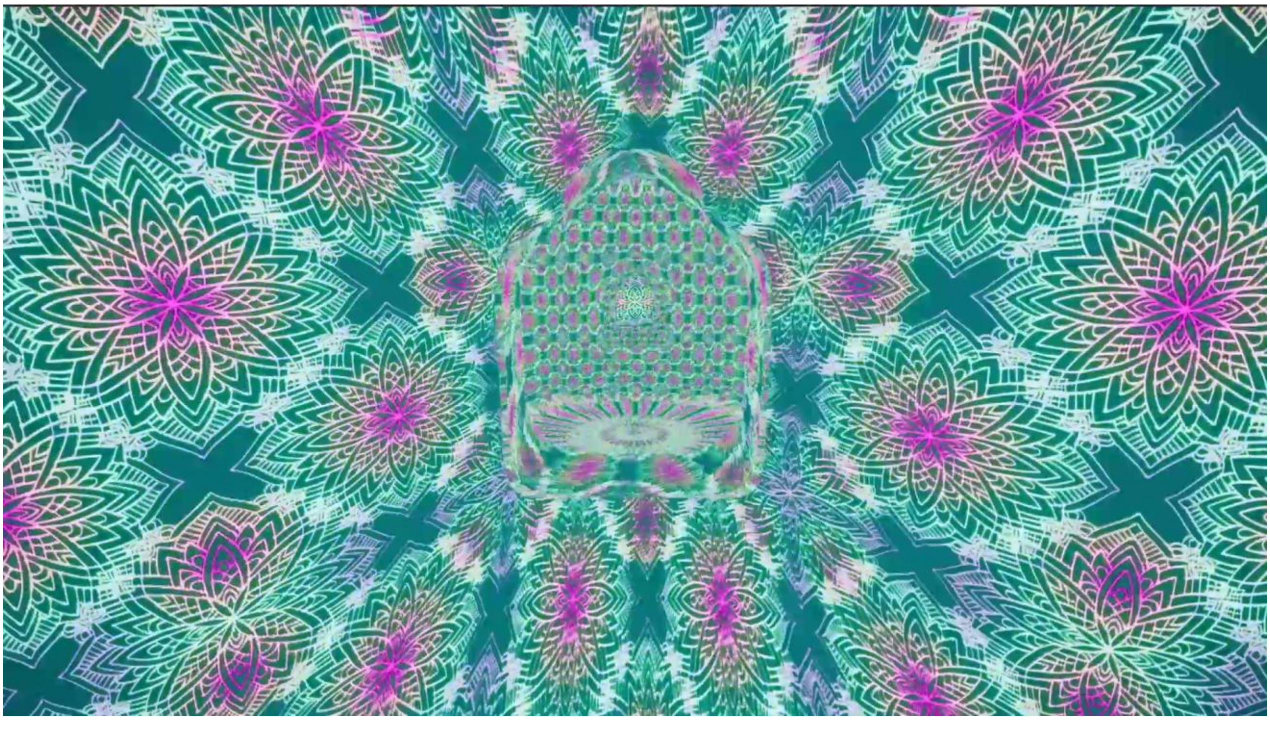

Figure S2. Screenshot of the DMT Hyperspace level which is also inspired by infinitely repeating, patterned cathedral-like structures sometimes reported in DMT experiences.

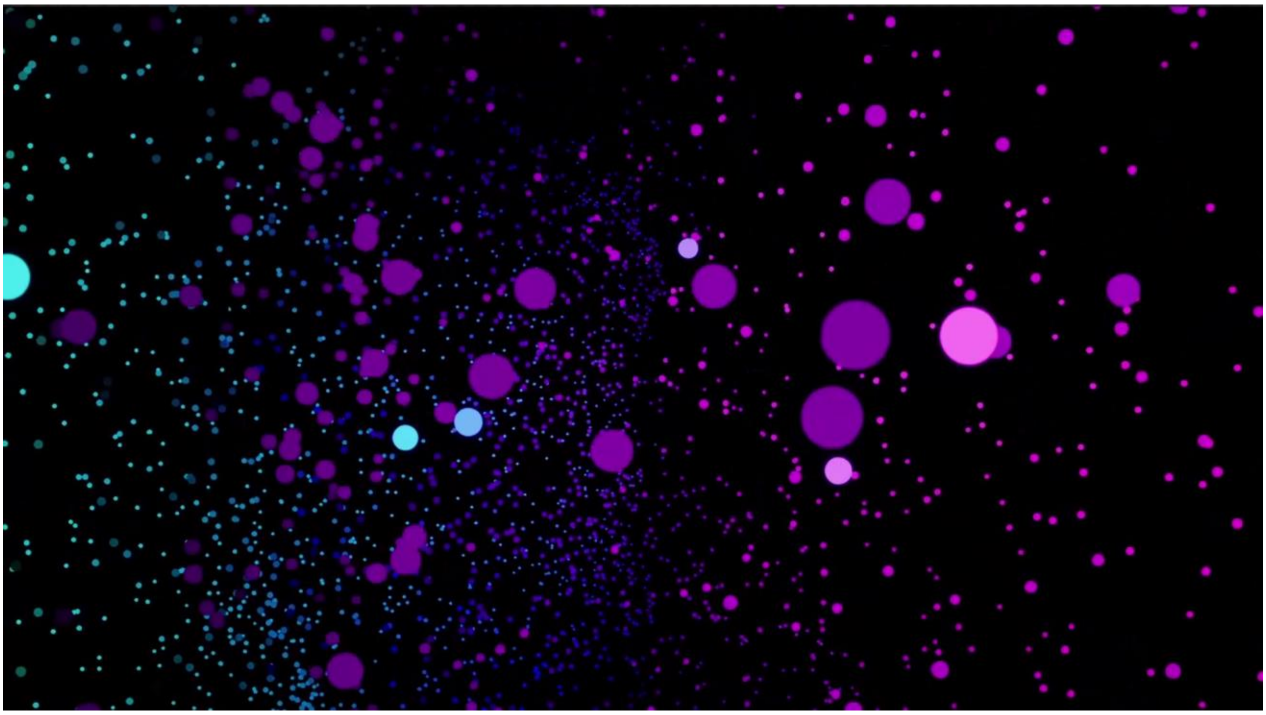

Figure S3. Screenshot of the Organic Life level. Here, some particles are introduced that circle around the player and change colour depending on the proximity of a few dominant particles, thus mimicking a sort of herd behaviour and creating a sense of interaction with the environment.

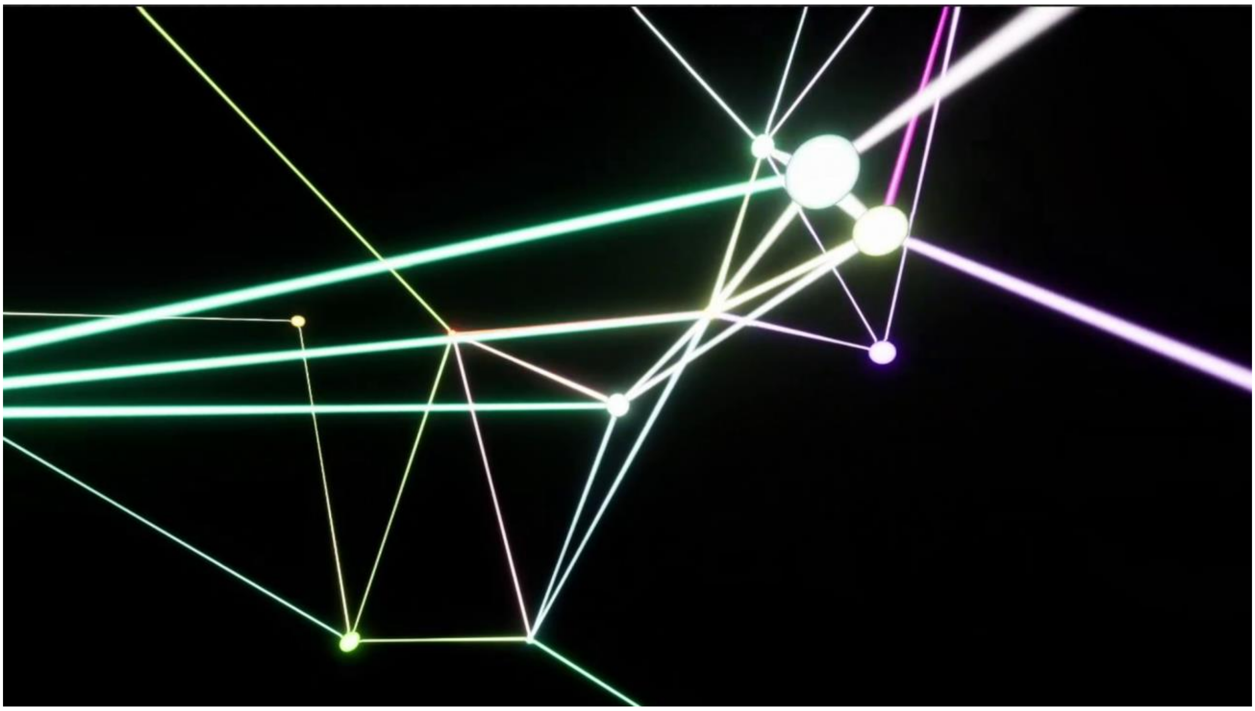

Figure S4. Screenshot of the Connectome level which consists of various rotating spheres and lines that dynamically connect nearby spheres and form random constellations, symbolizing the connection between all things and semantic networks.

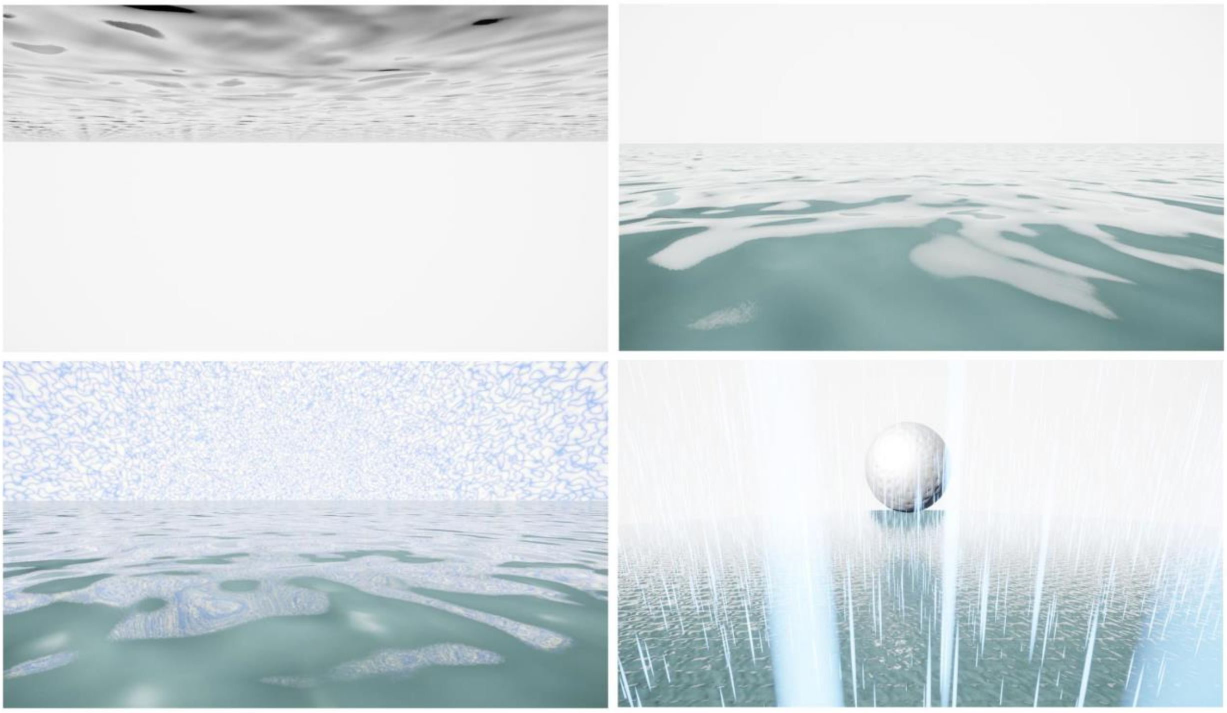

Figure S5. These screenshots represent the sequence of events in the Ocean level. Here the player first finds themselves under water, and then ascends above the waves. The sky in this level is pure white that gradually turns into an intricate pattern. After some time, a textured orb appears and the player is raised high above the ocean, with the water body below starting to glow.

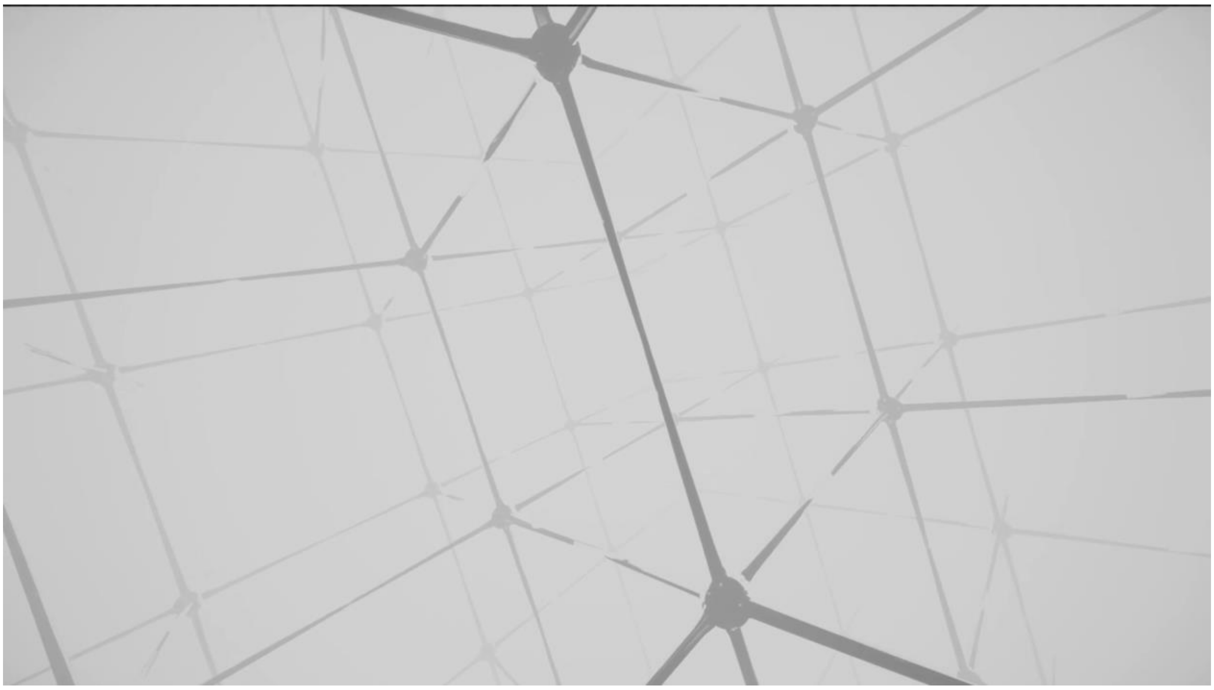

Figure S6. Screenshot of the Indra's Net level which illustrates an infinite net of connected hubs. Here we placed a custom shape with six arms in the middle of a previously displayed infinity level, thus creating the illusion of an endless net. In another implementation, we used the same shape in a particle system where particles were placed in a large grid formation and the player was moved through the net. A heavy fog was added to mask the outer edges of the net, thereby enhancing the illusion of infinity.

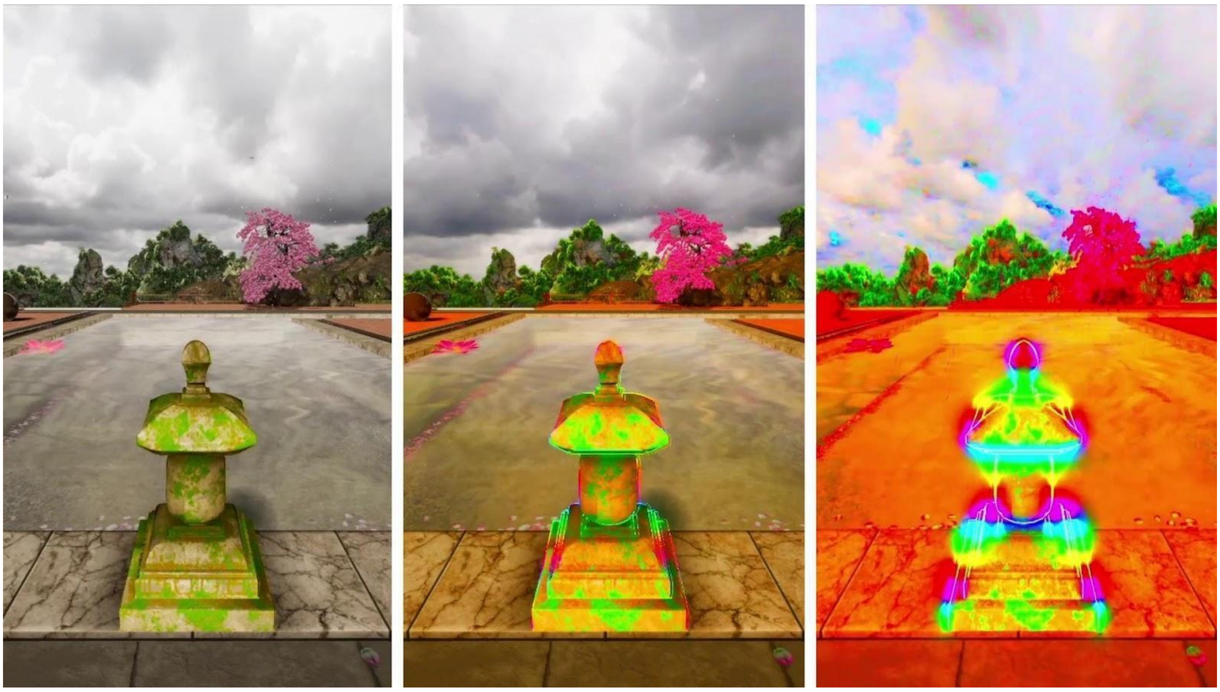

Figure S7. Gaze activated effects in the main level. Left - slightly increased contrast on the stone statue that the user is focusing on. Middle - increased contrast and edge aura on the stone statue, with effects mildly spilling over to the surrounding scene. Right - Strong contrast, edge auras and texture drifting.

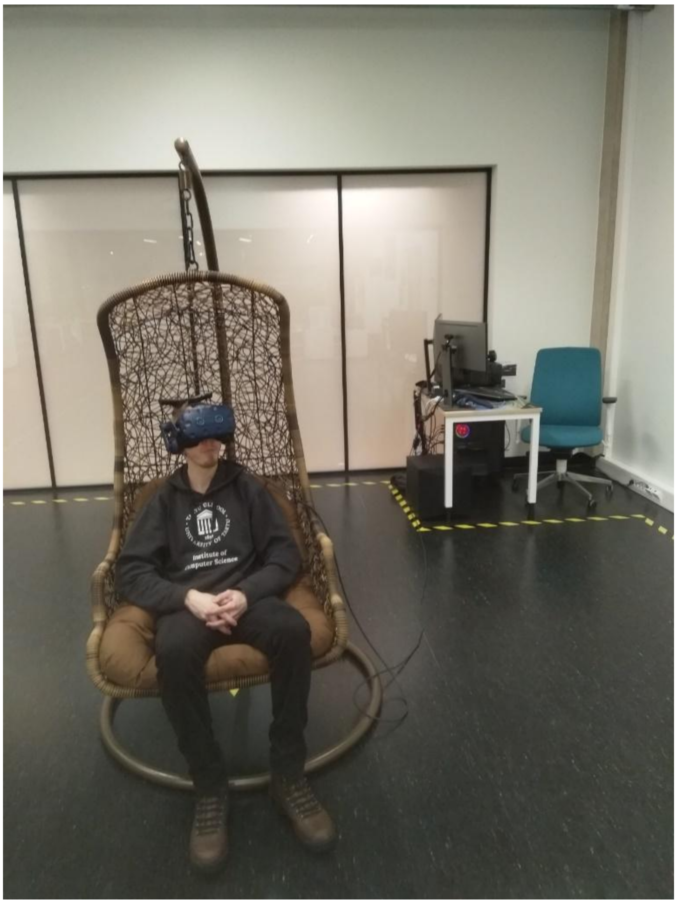

Figure S8. The environment where the experiments were carried out.

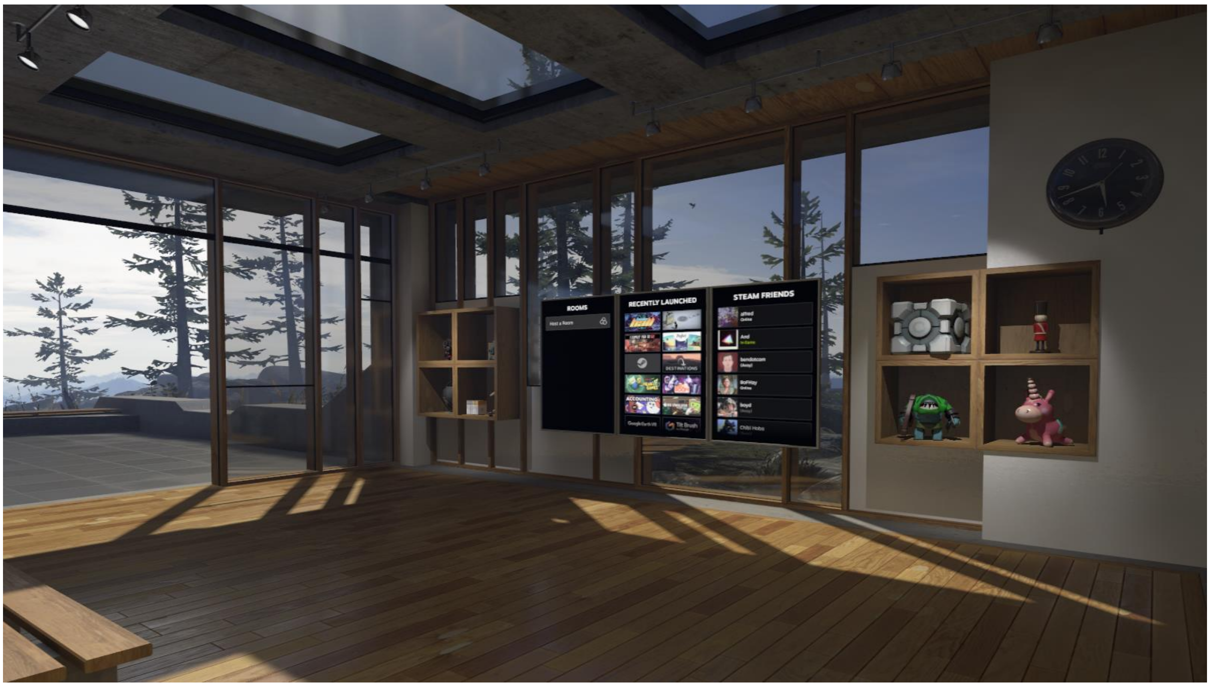

Figure S9. Default SteamVR Home environment[1] which was used for baseline measurements on the first day of the therapeutic intervention.

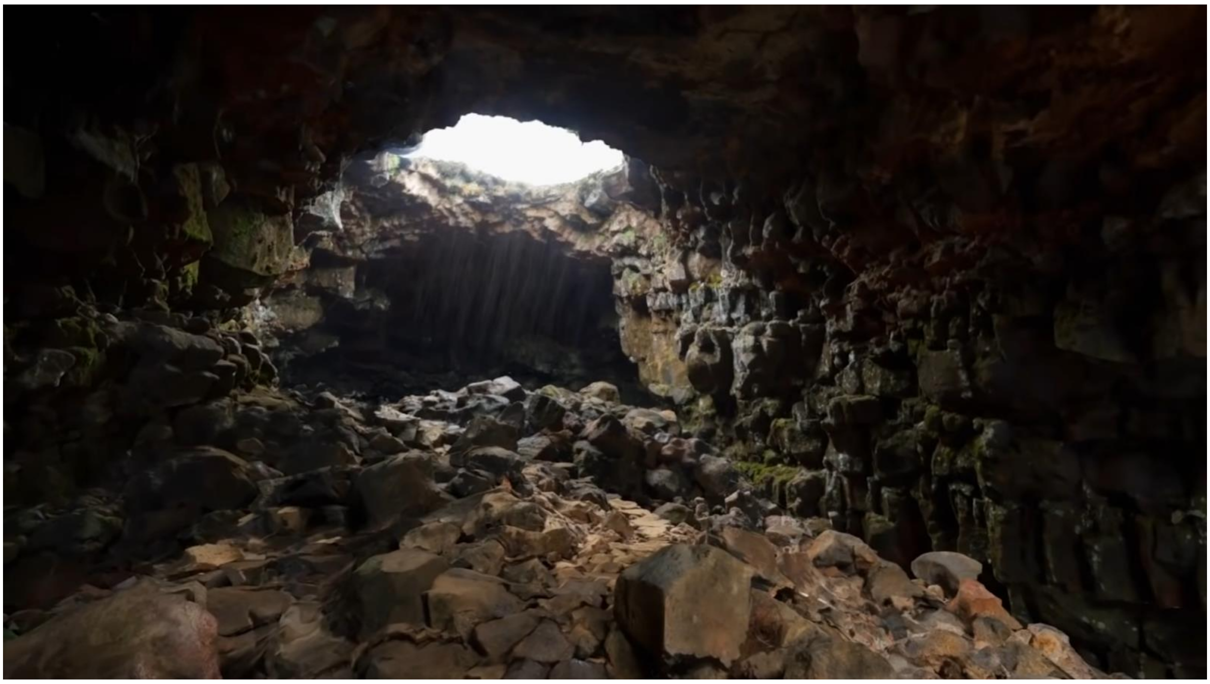

Figure S10. A cave-like environment which was used for the guided meditation on the first day of the therapeutic intervention[2]. It is, however, important to note that the participants were instructed to close their eyes for most of the time spent in this environment.

SM2. Additional EST-Q2 analyses

Additional stricter analysis of depressive symptoms

For a stricter analysis of the depressive symptoms we also removed two subjects (P3, P13) where we suspected that they had visited a psychiatrist or a psychologist during the follow-up period.

EST-Q2 depression subscale

The results stayed largely the same for the EST-Q2 depression subscale, decreasing from baseline (M = 15.20, SD = 2.95) to M = 11.22 (SD = 3.70) at follow-up ($t(8) = 3.02$, $p = 0.016$, Hedges' g = 0.932).

EST-Q2 anxiety subscale

The omission of two subjects also yields similar results on the EST-Q2 anxiety subscale with scores decreasing from baseline M = 9.11 (SD = 4.88) to M = 6.70 (SD = 4.71) at the follow-up ($t(8) = 3.30$, $p = 0.011$, Hedges' g = 0.394).

Additional analysis of depressive symptoms including all participants

We also conducted additional analysis based on an intention-to-treat approach including all the participants who finished the study (does not include P7, who did not answer the follow-up).

EST-Q2 depression subscale

The results on the EST-Q2 depression subscale decrease from baseline M = 14.67 (SD = 2.74) to M = 10.17 (SD = 4.00) at the follow-up ($t(11) = 4.180$, $p < .001$, Hedges' g = 1.106). The response rate was 25% and the remission rate 63,6% (as the scores of P12 were found to be below the cut-off at the re-evaluation of depressive symptoms during the first day of experiments they were excluded in the calculation of remission rates).

EST-Q2 anxiety subscale

The results on the EST-Q2 anxiety subscale decrease from baseline M = 9.33 (SD = 4.58) to M = 6.75 (SD = 4.24) at the follow-up ($t(11) = 4.531$, $p < .001$, Hedges' g = 0.585).

Additional analyses of MEQ, PIQ and EDI scores including all participants

For additional preliminary assessment of the effects of Psyrreal we also did analyses of MEQ, PIQ and EDI without excluding any participants. As the effect of a VR experience on these questionnaires could be relevant to some researchers also regarding a more general population than our, we also included additional analysis of these questionnaires including all participants.

MEQ30

MEQ30 average scores increased from M = 44.30 (SD = 21.33) to M = 49.13 (SD = 22.96), but the change was not significant (paired samples t-test: $t(12) = 1.173$, $p = 0.132$, Hedges' g = 0.186). Two participants (P6, P7) reported a complete mystical experience (all MEQ30 subscale scores over 60) after the demonstrative VR on day one. Three participants (P3, P6, P12) reported a complete mystical experience after day two. Interestingly, P7 reported a mystical experience after the demonstrative VR experience, but not after Psyrreal. The scores of P12 were close to a complete mystical experience even on the first day (narrowly missing out on one subscale).

PIQ

PIQ average scores increased from M = 28.03 (SD = 24.02) to M = 35.12 (SD = 24.70), with the change not being significant ($t(12) = 1.685$, $p = 0.059$, Hedges' g = 0.249). 9 out of 13 participants reported increased PIQ scores after day two. 3 participants reported an increase of over 20 points. 6 participants reported relatively high scores (30 or more) on day one, with 3 participants reporting scores over 55 points.

EDI

EDI average scores increased from M = 35.47 (SD = 19.70) to M = 45.92 (SD = 29.48), with the change not being significant ($t(12) = 1.746$, $p = 0.053$, Hedges' g = 0.357), 8 participants reported increases in EDI score after Psyrreal with 4 participants reporting increases of over 25 points.

SM3. Validation of Psyrreal

The sample consisted of seven participants with previous psychedelic experiences. The participants were recruited through social media and personal contacts. All participants had normal or corrected to normal vision. Additional criteria for exclusion were: hypersensitivity to motion sickness, a diagnosis of psychotic disorders and schizophrenia, a history of epileptic seizures or psychotic episodes, or a family history of schizophrenia.

The participants were asked to evaluate the similarity of parts of Psyrreal to psychedelic experiences. After each level, the participant was prompted in virtual reality to answer how similar the preceding level was to their psychedelic experiences on a virtual rating scale. They were directed to base their answers on the psychedelic experiences that they remember most vividly. As psychedelic experiences have massive inter-, and even intraindividual (i.e., between separate dosing sessions) variance, participants were also guided to generalize and extrapolate based on their previous experiences and try to imagine what could be possible in a psychedelic experience. Participants separately evaluated the visual similarity and the similarity of physical sensations induced by Psyrreal on a scale from 0 (not similar at all) to 5 (completely identical).

The ratings of the participants varied widely on both scales. One participant had an average score of 1.2 (SD = 0.77) and another an average of 4.15 (SD = 0.88) on the physical scale. On the visual scale, one participant had an average of 1.5 (SD = 1.36) while two participants had an average of 3.70 (SD = 0.92 and 1.03). The visual average rating across sample was 2.46 (SD = 0.92) and the physical average rating was 2.54 (SD = 0.92). In general, these results suggest that Psyrreal induces similar physical sensations and visual experiences as psychedelic compounds for some people.

Visual validation

Based on their visual similarity, seven levels were rated with the highest rating of 5 by at least one participant and a further 10 levels had a maximum rating of 4. Nine levels had a mean rating of over 2.5. Most levels had very contrasting evaluations by different participants with a range over 2 for sixteen levels, over 3 for nine levels and over 4 for three levels. Two participants had an average rating of 3.7 (Mode = 4, SD = 0.92 and 1.03) and one further participant had an average score over 2.5 (Mean = 2.65, SD = 1.18). Five participants rated at least one part with a 4 with two participants giving a rating of 5 to at least four levels.

Physical validation

Nine levels were rated with 5 by at least one participant and a further nine had a maximum rating of 4. Ten levels had a mean rating of over 2.5. Again, there were widely different ratings by different participants, with the range of ratings over 2 for sixteen levels, over 3 for 10 levels and over 4 for one level. Two participants had an average score of over three with further two having an average score over 2.5. Six participants rated at least one part with a 4 with two participants giving a rating of 5 to at least four levels.

SM4. Thematic Analysis

The following includes the stand-out themes and corresponding excerpts from the transcripts of the integrative discussion after experiencing Psyreal. The excerpts are translated from Estonian. Participant 6 was excluded from the analyses due to receiving antidepressant treatment during the intervention and participant 12 was excluded due to reporting scores below the cut-off on the EST-Q2 depression subscale, but their excerpts are included in the following document.

The questions asked in the semi-structured interview were:

- Did you experience any insight or did you understand something of personal relevance or fundamental? Did you find answers to your questions?
- Did the experience have an effect on your conscious state? If so, how would you describe the change?
- Did something prevent you from being completely immersed or cause you to lose immersion during the experience? If so, what caused the loss of immersion?
- Did you experience any alteration to your self-image or loss of a sense of self? Or any alteration or dissolution of your physical sense of self?
- Did you experience any emotions? If so, what kind of emotions?
- Did any part of the experience make you think? About what?

<u>Somatic effects – 4 (P1, P5, P7, P10)</u>

“When it [the experience] was over then I realized I was suddenly very hot and a wave of heat went through my arms” (P1)

“I could feel this movement. There’s [this part] where you’re moving or feel like you’re moving and everything is rotating. That was fascinating. In the back of my brain I felt something activate and intensify, that was cool.” (P5)

“Then suddenly I had tactile sensations. At the tip of my fingers, I started to feel structures. […] There was this pattern of a carpet [sic!] and it was soft. **So you felt the pattern of a carpet?** Yes, I had the carpet on the tips of my fingers. It was like a cozy colour and it was soft. Basically, the pattern exuded so much softness.” (P6)

“[…] Okay, but relax now, go back into this experience. Then I went into the experience and I had physical reactions at some point.” (P7)

“At some point [I felt] something in my upper stomach, like the muscles tensed up. […] I was thinking whether it is because of how I’m sitting in this chair, or is it some kind of a tension. But I didn’t get any answer to this – this tension sort of dissolved away at some point.” (P10)

“These tunnel parts like pulled me with it. I felt it was very strong, it also caused like a physical reaction.” (P12)

“[…] The experience did affect how I felt my body, but it didn’t disappear, no.” (P5)

<u>Made me think – 5 (P3, P5, P7, P11, P13)</u>

“[The parts] with the landscape and waves and rays did not make me think as much. The [more abstract] parts with the patterns made me think more.” (P1)

“I had a lot of different thoughts in my head at the same time.” (P3)

“At some points my mind did start to wander and I didn’t focus on the experience. **What do you mean by mind wandering? Did you start thinking about some mundane things or?** I mainly started to think about the questions that we wrote down before. Mainly about those [questions]. I didn’t have any random mind wandering.” (P5)

“I thought about the symbolism quite often. Or I was trying to not think about the symbolism, but I still thought about the symbolism. [I was] wondering what all of these things [stimuli in the experience] mean.” (P5)

“**Did you have any thoughts about your questions?** Yes.” (P6)

“**Did some parts of the experience make you think more? You already described some parts that made you think. Do you remember anything else?** Yes. These [abstract] patterns. [...] [When I go to bed] these patterns start to play [in my mind] and then after looking at these patterns I fall asleep. ” (P6)

“I really went into the experience. I didn’t start to think about any questions – I just let the experience guide me. And then at some point my thoughts started racing. […] I really let this experience carry me and I went with it. I didn’t think about anything at the start, I just sort of ‘was there’. And then at some point these thoughts started appearing.” (P7)

“**Did any part of the experience make you think more?** Yes, if you ask like that. Maybe these large empty visual areas or the parts that resembled space or the universe made me think more.” (P8)

“**Did any part of the experience make you think more?** Yes, the temple [Main level] made me think, because you can just be calmly and think. Think about nature and other things, and just be […]” (P9)

“You observe and then some thoughts emerge. Then something changes and some new thoughts appear.” (P11)

“**So you thought about the previous [questions] during the experience?** To some extent, I made some connections.” (P11)

“From getting in touch with this emotion, all these other questions also started to unravel themselves, and I understood several [cognitive and behavioural] patterns.

“I did have more impulses or things to think about [than usual].” (P13)

<u>Changes in perspective - 5 (P2, P3, P4, P5, P7)</u>

“Maybe it gave or reminded me that the universe is much larger than me with my little problems.” (P2)

“[I had] like an “overview” feeling. Especially when there was this big dark space around me, or a view of the Earth. There I had the feeling as if I was like a small dot somewhere, like… It’s hard to describe. […] When there were stars all around, it was like every one of us is like a little star.” (P3)

“Like a very profound feeling. […] Like being a part [of a larger whole], yet not a part of it – like a dissonance.” (P3)

“[…] Observing more so the whole than the details. There was this shift in perspective that in the beginning, I was looking more at the details, and afterwards, I was looking more at the whole.” (P3)

"I'm so small, that all [my] problems don't have any relevance. It puts things into perspective. If you see the larger perspective, then you're not thinking about the smaller things." (P6)

"This is the same insight that I'm describing. I have like a different perspective in my consciousness. It's like there's space. Like an "aha", wonderment, or like "aha, okay". It's pretty awesome… Or very awesome. Maybe I can't describe it well, but I developed a different perspective of myself, "the self" in general and also of the therapeutic process." (P7)

"And, especially towards the end, these [patterns] became very clear. I used to think that it's [pattern of earning the love and approval of others] mainly related to my work-life, but I see that it is elsewhere as well: in my family-life, social life, everywhere. [I have] this pattern that I [have to] be good to earn the love or approval of others. This became very clear to me." (P12)

"This is quite random, but I was thinking that… I don't know why I didn't understand it then. When we were learning about the planet Earth in fourth grade, how did I not understand that these white spots were clouds. Now I was like, aha – it's the weather. […] It makes a lot of sense, but I just hadn't thought about it like this before. That the weather we have here is nearby – you don't have the weather in outer space." (P4)

Feeling of insight – 3 (P1, P5, P7)

"I had an understanding, but I can't put it into words. […] It was more like a feeling rather than imagination of something, but also not like one feeling but like… It was so trippy – kind of like a movie but without characters. But it's also not a movie, but just a feeling that is moving and it has a script, which is just the experience of that feeling. I had that kind of an understanding, but I can't put into words right now what I mean by it." (P5)

"Really like a physical experience, that yes, I can be programmed, that I can reprogram myself. Not go into therapy to heal "myself", but that this self doesn't exist." (P7)

"I have experienced this, where it's [sense of self] completely gone. That I'm one with nature or that everything is one. I have felt that very sharply in my life at multiple times. This wasn't like that, it was like a different kind of insight, like a new, physical insight. […] This insight right now, it's something that I'm experiencing here right now. It's something that I can take with me to every moment." (P7)

"Yes, I would say that I didn't find an answer, but I felt surprisingly much like it was a step closer to it [understanding]." (P1)

Intellectual insight / Understanding – 3 (P3, P5, P7)

"It's always been important to me that I have control over a lot of things, and sometimes this is counterproductive. **Okay, so you had this understanding?** Yes." (P3)

"**Did you experience insight or understood anything personally relevant or fundamental during this experience?** I understood something, yes. […] I understood that maybe I should take some steps that might be unpleasant, but will later lead to a better quality of life." (P5)

"I had an understanding […] that every day I'm new, every moment I'm new. Why am I holding onto or dragging this experience [traumatic event from childhood] with me? It is possible for me to reprogram this. [I had] this experience that we have this immense potential for change or we're always in the midst of all these forces. It was awesome, I haven't experienced something like that before, it was new for me. Actually, this understanding was completely new for me." (P7)

"**How do you feel, did you experience any insight or did you understand anything personally relevant or fundamental?** Somewhat. I had a lot of different thoughts at the same time." (P3)

“**Did you find answers to your questions or did you have any thoughts about your questions?** A little bit, yes. I think this process of thinking is still going on, I didn’t get to the end of it. But I did have this thought that everybody is trying so hard – maybe everybody is thinking like this.” (P3)

“[…] You are in this environment which seems very comfortable, but some things bothered me. It was gloomy. Then when it changed, [it was] like spring came and the sun came out and the sky turned blue, it was very liberating, that you understood that the environment affects so much how you feel in the centre of it all.” (P3)

“[...] I really went into the music. […] With the music I had a good idea that I now know what I’m going to do – I’m going to a concert with my son. […] *Out of the blue* [sic!]! If I like to listen to music and he likes it – let’s do something together, let’s just go.” (P6)

“**So you could say that you found answers to your questions?** Could say that yes. I am stronger now, today.” (P6)

“**[…] Did you find answers to your questions?** Yes. **Did you have any thoughts about your questions?** Yes. Like, what am I so worried about, that I have to do something.” (P6)

“I had something like an insight related to the [traumatic] experience I had as an eleven-year-old. I had the same feeling for a moment and I understood that maybe I’m not allowing myself to feel all of these feelings. […] It’s something that I’ve been numbing, I would need to slowly open myself to it. That’s the understanding, maybe. That I’m repressing my emotions. [...] I need to contact and accept these feelings of depression and hopelessness.” (P7)

“[There is no] finding that this is me or that this is my so-called true essence. Rather, I had the understanding that something like my essence doesn’t exist, that it is just some influences of influences, more like a decision or awareness or luck or chance. But now, by being aware, we can change this. […] This whole experience, when I was 11, I haven’t thought about it much at all. [I] acknowledged that it was traumatic. I think I will continue observing this [experience] in meditation. [I will try to work] on accepting and observing these feelings, not ‘going with them’, but observing, feeling – feeling them through, and becoming aware of them. So today, this new understanding that I can [re]program myself and that my ‘essence’ doesn’t exist, it is very liberating. It gives me, in some sense, a vitality that I’m looking for. I have this strength in me. This programming happens anyways, the question is whether I take control over the reins.” (P7)

“From getting in touch with this emotion, all these other questions also started to unravel themselves, and I understood several [cognitive and behavioural] patterns. And, especially towards the end, these [patterns] became very clear. I used to think that it [pattern of earning the love and approval of others] is mainly related to my work-life, but I see that it is elsewhere as well: in my family-life, social life, everywhere. [I have] this pattern that I [have to] be good to earn the love or approval of others. This became very clear for me.” (P12)

“As a solution I still saw that I have to fill up my calendar with the things that I want to do, and if vacancies remain, then I can allow these to be filled by the needs of other people. I can’t spend all of my time and life based on the needs of other people. This became very clear to me in the end [of the experience].” (P12)

“[…] It is a very strong automatic behaviour for me – to act based on other people’s needs. This and being good, became very apparent for me. **Specifically during the experience?** Yes, specifically, during the experience.” (P12)

“This is the same insight that I’m describing. I have like a different perspective in my consciousness. It’s like there’s space. Like an “aha”, wonderment, or like “aha, okay”. It’s pretty awesome… Or very awesome. Maybe I can’t describe it well, but I developed a different perspective of myself, “the self” in general and also of the therapeutic process.” (P7)

No novel insights - 6 (P2, P4, P8, P9, P10, P13)

"**Did you experience any insight? Did you understand anything personally relevant or fundamental?** No, I did not experience that." (P2)

"**Did you find answers to your question?** No." (P2)

"**Did you experience any insight? Did you understand anything personally relevant or fundamental?** Rather not. […] I wouldn't say that I found any answers really." (P4)

"I felt different emotions, and I had maybe made some different connections between some experiences, knowledge or facts. But, on a personal level, like an insight or kind of an experience [of understanding], rather not." (P8)

"**But did you experience any insight?** I would not say that I did [experience insight]" (P9)

"**But insight?** Like a deeper insight did not come. It might come tomorrow, or the day after." (P10)

"**Did you experience any insight? Did you understand anything personally relevant or fundamental?** Well, because I didn't really think about these things, then I think not. Rather not." (P13)

"**Did you find answers to your questions or did you have any thoughts about your questions?** I didn't have any [thoughts about my questions]. There wasn't even any connection to the questions. These questions are intellectual, but in meditation, or [in the experience] looking at these visuals, there is just so much more to do, and more interesting things to do than to deal with some personal intellectual juggling or something." (P10)

Ineffability - 4 (P3, P5, P10, P7)

"[I had] like an "overview" feeling. Especially when there was this big dark space around me, or a view of the Earth. There I had the feeling as if I was like a small dot somewhere, like… It's hard to describe." (P3)

"**Did you have any emotions? What emotions did you experience?** I did, yes, but it's very hard to explain them. I became emotional, but I didn't really understand why." (P3)

"At first there was this meditation, which was very calm and logical. And then at some point, you had this thing [experience] that is tremendously hard to describe or even grasp what is going on." (P3)

"I had an understanding, but I can't put it into words. […] It was more like a feeling rather than imagination of something, but also not like one feeling but like… It [the understanding] was so trippy – kind of like a move, but without characters. But it's also not a movie, but just a feeling that is moving and it has a script, which is just the experience of that feeling. I had that kind of an understanding, but I can't put into words right now what I mean by it." (P5)

"Infinity. I've tried to verbalize it – it can't be put into words. It can only be imagined. Or it can't [be imagined], I don't know. I can't describe it." (P10)

"It got so fast for me, that physically it seemed to be impossible to move so fast. And it caused this confusion. It made me feel that this can't really be and at that moment I felt like a dissolution or something. Because it seemed like such an impossible feeling, then this boundary [between the body and mind] disappeared. That's how I can describe it. (P12)

"This is the same insight that I'm describing. I have like a different perspective in my consciousness. It's like there's space. Like an "aha", wonderment, or like "aha, okay". It's pretty awesome… Or very awesome. Maybe I can't describe it well, but I developed a different perspective of myself, "the self" in general and also of the therapeutic process." (P7)

Alterations in embodied sense of self – 4 (P1, P2, P3, P5)

"**So you are saying that you experienced losing a sense of self or a dissolution of the physical self?** Yes." (P1)

"**Did you experience a loss of self-image or sense of self, or dissolution of or alteration to your physical self?** I would say yes, but a little bit. I mean, I did not feel the loss of my self-image, but the other things [I did feel] a little bit. At some points it was as if I had forgotten that I'm sitting here. Or like [my sense of self] disappeared a little, but I'd say that during yesterday's meditation, I also, bizzarely, experienced it a bit. That at one moment you discover that, aah, I'm sitting here." (P1)

"**Did you experience you're an loss of self-image or sense of self, or alteration to your physical self?** Yes, like a dissolution, yes. […] The experience was so immersive, that you melted into it." (P2)

"Not like a loss, but the experience did affect how I experienced my body. But it didn't disappear, no." (P5)

"[…] It's the same, that you don't understand if you've moved or the screen has moved or the view has moved. This sense of space was lost. Maybe there were some moments where the sense of my body was also gone – at one point you didn't feel your hands and then suddenly they were there. But this wasn't stable, but it happened every now and then." (P5)

"At some moments, I was startled by my hands, by [becoming aware of] feeling my hands. So I presume that at some point it [sense of physical self] was gone, but I can't consciously remember it. I think if I would've consciously [experienced] it, then I would've brought my sense of self back. I presume that there was some amount of loss [of my sense of self]." (P5)

"**Did you experience you're an loss of self-image or sense of self, or alteration to your physical self?** […] Yes, I did. It was physical." (P6)

"**Did you experience a loss of self-image or sense of self, or alteration to your physical self?** Yes, it was at these moments when there was this tunnel and these dots." (P12)

"It got so fast for me, that physically it seemed to be impossible to move so fast. And it caused this confusion. It made me feel that this can't really be and at that moment I felt like a dissolution or something. Because it seemed like such an impossible feeling, this boundary [between the body and mind] disappeared. That's how I can describe it. (P12)

"**Did you feel alteration to your physical self, or dissolution?** […] A little bit yes, but it was a little bit disruptive that these glasses were actually quite heavy. You feel this pressure occasionally that brings it [the sense of self] back a bit, but generally I did [experience alteration to the physical sense of self]." (P3)

Alterations in narrative sense of self – 3 (P3, P7, P9)

"When I looked down, I didn't see myself and I had like a feeling of floating. And [a sense] that the "I" is not so important – I didn't think about what I'm like. I could really focus on what everything else is like." (P3)

"[I] had a question about the self. **What is the self?** Yes, what is the self. I'm not talking about the level of, being one with everything or some "*ultimate truth*" [sic!]. But I'm talking about this construct named [P7]. These voices, influences, childhood patterns – how much are these things [me]? That was awesome and balanced out the dread [related to the memories of her childhood trauma]. I had this recognition that I can reprogram myself." (P7)

"**Did you experience loss of your self-image or sense of self?** In the temple [level] a little bit, yes. **You can describe it a little.** If everyday problems disappear, then you also disappear, because you are at the focus of these problems, usually. But in a calm state, you have time to

think about nature and other phenomena that are unrelated to you. In that sense you disappear, this egocentricity disappears. But these problems don't ever disappear completely." (P9)

"Well, at some point I felt a great connection to the living. I think it was the moment where you went through these dots. I had this very strong feeling of losing the boundaries of the body and the soul. It was a very positive feeling." (P12)

"[…] What others think of me – it became less important." (P3)

"[…] You are in this environment which seems very comfortable, but some things bothered me. It was somehow gloomy. Then when it changed, [it was] like spring came and the sun came out and the sky went blue, it was very liberating when you understood that the environment affects so much how you feel in the centre of it all." (P3)

"[It was] truly a physical experience [understanding] that I can be programmed, that I can reprogram myself. Not go into therapy to heal 'myself', but that this 'self' doesn't exist." (P7)

Technical issues (Breaking immersion) – 3 (P1, P3, P13)

"At some points, there were some connection issues with one of the headphones." (P1) * NOTE: This was fixed after the first experimental session with 3 participants.

"The left headphone started to crackle when I touched it. This broke my immersion for a moment." (P3)

"These glasses are actually quite heavy, so you feel this pressure occasionally. This also broke the immersion a bit." (P3)

"Maybe that I couldn't support my head. You have this lump at the back [of the glasses]. [I] would've wanted to support my head, but it wasn't possible. This was perhaps the only physical thing that [broke the immersion]." (P12)

"One technical thing that I noticed multiple times – I would've liked it to be louder." (P13)

Loss of VR presence (Breaking Immersion) – 5 (P1, P4, P5, P10, P8)

"I'd say that a large part of these visuals were clearly man-made." (P1)

"[…] When you start to fall [virtually], then you immediately start to […] check your other senses [whether you're falling or not]. […] But this drew my attention away from the [experience]." (P1)

"I felt that it took quite a lot of energy for me to stay calm. When the colours were moving very fast and the patterns or the waving, it took a lot of energy. […] I couldn't think about anything else and my attention was focused on that [the fast moving stimuli etc]. […] This feeling of discomfort took up a lot of my attention." (P4)

"I was quite actively aware that this is not real. You have this physical sensation of having something on your head and your head is heavier. I supported my head as well at some moments. My neck got very tired." (P4)

"And our field of view is usually larger. This created a strong sense of like watching a movie – I was not really there." (P4)

"[As] I also mentioned yesterday, the quality was not as good as my vision. I have pretty good vision. So it felt a bit like a computer game or a movie."

"**Did something stop you from being completely immersed during the experience, or did something cause you to lose immersion?** I did start thinking about some of my thoughts. This might've affected the direct experience of it, but, on the other hand, it was

indirectly the point of it – to connect it to my own thoughts. At some points my mind did start wandering and I didn't focus on the experience." (P5)

"I consistently had the feeling that this is something that's coming from the outside. A meditative state generally starts from the inside – it comes from inside out. Here [there were constant external stimuli], so you don't have these quiet moments where you can go deeper." (P10)

"It also lasts quite long. You get a bit tired just sitting in one position." (P12)

"If I wasn't so tired, or maybe if today's experiment would've been earlier, I might've been able to get into [the experience] more. Being tired didn't let me enjoy it completely or let go." (P8)

"**What do you mean you lost your attention?** I lost my attention in the sense that I was thinking: "what's next?" […] Some things were not that interesting. **So you're thinking that you weren't present there?** [I was not] present, yes.

<u>Importance of letting go – 2 (P3, P7)</u>

"[…] If there was simultaneously very intense music and visuals – [this caused] a very strong feeling - then I understood that it's smarter or easier to let go at those moments. Without trying to think about where you are or what you are doing, just going along with the movement […]" (P3)

"In the beginning I wanted to maintain control or keep holding onto something. I was very specifically focused on the details. But when I let go of this, it became much easier." (P3)

"If there is movement that is not of your volition, you keep trying to hold onto it and look at individual details. You can observe every detail, but in the end it wasn't that important. Rather, [it was important] that you're there, in the moment, or in that emotion. [It was important] that you let that control go a bit." (P3)

"There was this feeling of letting go– you're so used to desperately holding onto everything and then [I felt] this relaxation." (P3)

"You can't always control everything. And if you sometimes do try, it gets worse." (P3)

"[During the experience] I was waiting the whole time for something to happen, then it happened by itself, after I stopped waiting [for it]." (P6)

"I really went into the experience. I didn't think about any questions – I just let the experience guide me. And then at some point my thoughts started to run. […] I really let this experience carry me and I went with it. I didn't think about anything at the start, I just sort of 'was there'. And then at some point these thoughts started to emerge." (P7)

"[…] and then at some point I let go, thinking that it [the experience] doesn't work like this. **And when you let go?** Then it actually started working, yes." (P12)

"This visual and auditory intensity of going down some pipe or into some funnel was very intense for a moment. The first time was extremely intense, but it helped to focus more on the feeling, not on what you're seeing." (P3)

"Maybe about half of it, I'd say, I was holding on, or thinking that this [experience] is trying to manipulate my brain. I had a very strong sceptical feeling. […] I had asked this question about my sadness. And then at some point I felt very strong sadness. Or I went into this place of very strong sadness. And that was the moment when this experience actually started working for me. From getting in touch with this emotion, all these other questions also started to unravel and I understood quite many of my [cognitive and behavioural] patterns." (P12)

<u>Sadness – 4 (P3, P4, P5, P7)</u>

"At some points I felt sadness as well." (P3)

"In the end I felt a bit of sadness, but I didn't understand why I felt it. But I was a little bit sad." (P4)

"And then at one point I felt really strong sadness, that was when there were these lines [Mystical Light level]. There was this one bright star and then there were lines everywhere, that made me incredibly sad."(P5)

"[...] Well, the music made me sad. I really love the violin and I thought, okay, here come the tears [...]. Then I remembered how I listen to music and that I should do it more, that emotion was very strong." (P6)

"[…] I think it was like a suicidal depression. I experienced it." (P7)

"This feeling of loneliness. A feeling of being left without, a feeling of loneliness. I don't remember the thoughts with it, but rather the feeling of it. Loneliness and sadness." (P12)

"It's interesting that this sadness, I'm kind of okay with it now – it's part of the experience. I had it [the sadness] at some moments, but it is a little different from usual." (P3)

Anxiety - 1 (P1)

"For a moment there was this anxiety as I suddenly remembered that I have often dreamt that I'm basically like falling or something, there were several scenes where it's as if you're in free fall. The first emotion that I felt then was fear and anxiety […]." (P1)

Fear – 4 (P1, P7, P8, P13)

"For a moment there was this anxiety as I suddenly remembered that I have often dreamt that I'm basically like falling or something, there were several scenes where it's as if you're in free fall. The first emotion that I felt then was fear and anxiety […]." (P1)

"Dread, with this same one scene [Mountain level] I had a feeling of dread." (P7)

"I must admit that this [suspended] chair caused a lot of stress or anxiety for me yesterday. I thought that I'm gonna sit in it and fall down [break the chair]. This was also therapeutic, in a sense – I overcame that fear. I thought that it [feelings of anxiety] doesn't matter – I'm gonna sit there [in the chair]. At first it was like okay, [acknowledging] this is fear – I'm just going to sit. Then today I had the same feeling for a moment, and I thought that it doesn't matter, even if I fall down, then it's their problem." (P7)

"One emotion that I can recall which was quite strong - for a moment I even thought that maybe I should remove the [VR] goggles - was this intensity or also a bit of fear when there was this visually very big change from that first [environment] and where the visual shapes started to "run". Just before, what could have brought this feeling of fear was that when I was looking at the clouds that had different strange things happening to them, the colours were changing and so on, then I had this apocalyptic feeling or like the world was ending, that is this what the end of the world could be like. And related to that I felt- maybe that's where the feelings of fear began, this loneliness and fear. [And I was thinking] that maybe this solitude is the end of the world for me." (P8)

"All of this [sensations of movement during the experience] was very intense. Almost so intense that you want to hold your arms out in front or you are afraid of falling against something. It was almost so strong that I would've liked to scream a little bit." (P13)

Boredom – 3 (P4, P9, P10)

"When there were these patterns for a while then I got bored. And tired. Like if you're at the cinema for a long time, and it's dark and the movie isn't very interesting, then you get tired. This kind of boredom." (P4)

“The part with the abstract shapes, that was quite boring. This kind of art has never touched, impressed or interested me.” (P9)

“Is boredom also an emotion? I felt that too. It was more exciting in the beginning, but at some point things started to repeat itself or became a little bit annoying.” (P10)

Tiredness/Sleepiness – 6 (P4, P5, P9, P11, P13, P8)

“The tiredness was rather quite general. I don’t know, maybe it’s because I’m generally tired. These dark environments at some points encouraged falling asleep.” (P4)

“I got very sleepy in the end. Terribly sleepy. I was so tired that I almost fell asleep.” (P5)

“Exactly during the experience I had a very strong bout of fatigue.” (P9)

“[I feel] a little bit tired [right now]. I got sleepy [and I] would’ve liked to move around at some points.” (P11)

“It also lasts quite long. You get a bit tired just sitting in one position.” (P12)

“**How are you feeling right now?** As if I just woke up. I might have dozed off a bit there in the middle of it [the experience].” (P13)

“If I wasn’t so tired, or maybe if today’s experiment would’ve been earlier, I might’ve been able to get into [the experience] more. Being tired didn’t let me enjoy it completely or let go.” (P8)

Cybersickness / Nausea – 4 (P4, P5, P8, P10)

“I feel a bit poorly. I had a bit of nausea from the spinning [tunnels].” (P4)

“I had slight nausea for a moment, but actually going into [the feeling of nausea] makes you understand that it’s not nausea but just confusion of the senses – going out of balance.” (P5)

“That’s when I felt this intense feeling, as if it was pulling me into it somehow, and also [I felt] a little dizzy. But maybe it was just the first time. Afterwards, I got used to it and I didn’t get that feeling anymore.” (P8)

“In the beginning I had a feeling of being drunk or when you go to sleep while drunk. Once your head hits the pillow and you close your eyes then that’s the exact same feeling - a carousel. I don’t know if that’s an altered state of consciousness, I guess it is. That was in the beginning. But then at one point I got used to that visual and the movement and then I no longer got that feeling.” (P10)

Comparison to games/movies – 5 (P9, P10, P11, P4, P8)

“At some point there was like a fortress in the sky. If you could’ve moved around there, to draw a parallel with computer games, that would’ve been more interesting than those abstract shapes.” (P9)

“Compared to computer games, this world is not very interesting. In some computer games you have very large worlds. And here, if you just have one temple where you can’t move, then it doesn’t really compare. Even in movies and such, or in the real world, you can move around. In that sense, it was a little bit boring – being able to interact adds a lot.” (P9)

“I remembered all these things – science fiction of the 80s, stories from people and my own experiences of visions and [similar] things.” (P10)

“Like a computer game feeling. Something that is quite close to things in the real world, but something is wrong or missing – not quite ‘it’. When the shapes came, then [it felt] very much like a computer game.” (P11)

“ [Compared to] everything else that is related to visual arts or what’s presented on a screen, I’ve never experienced them from so close, so it was a very strong experience. I’ve been to panoramic cinemas and all kinds of things in England and all over Europe – like skywatching and all kinds of things. But [this feeling] has never been so strong.” (P6)

“[As] I also mentioned yesterday, this quality is not as good as my vision. I have pretty good vision. So it felt a bit like a computer game or a movie.” (P4)

“[I made] some connections to sci-fi movies that I’ve seen and some games that I’ve played. Auditory as well as visual connections. Some things reminded me of things that I’ve seen [in computer games or movies].” (P8)

Feelings of calmness/peace – 6 (P1, P2, P3, P4, P8, P9)

“At one point I felt content. I’m not sure if that is the best word for it. For lack of a better word, I’d say it was *chill* [sic!].” (P1)

“**Did the experience affect your conscious state?** Definitely, yes. It calmed me down, for example.” (P2)

“At some points I had this feeling of peace.” (P3)

“At those moments when the world around stood still, then it felt calm […].” (P4)

“I felt that everything wasn’t moving and changing there [in the Main level] all the time. So I could just be calm, it was like a safe feeling.” (P4)

“[Visual geometric] patterns calm me down so much. […] [When I go to bed] these patterns start to play [in my mind] and then after looking at these patterns I fall asleep.” (P6)

“Maybe these large empty visual areas or the parts that resembled space or the universe [made me think more]. As they were less intense for me, then they made me feel more calmness than these very fast and changing visual experiences.” (P8)

“The music and the temple [Main level] with the meditation is calming enough to allow me to focus and be interested [in the environment].” (P9)

“A good feeling. Generally a very positive or peaceful feeling.” (P12)

Feeling of joy/pleasure – 6 (P3, P5, P10, P11, P7, P13)

“At some moments I had a very joyful and liberating feeling. When you got out of these very strong intense scenes into calm and quiet environments then it made me very happy for a moment.” (P3)

“This was in the beginning, when everything turned into a LSD trip. I don’t know if that made me laugh, but it was a lot of fun.” (P5)

“Gratitude! Complete joy and gratitude. Really, as if I’ve just been to the cinema and psychotherapy as well. A completely therapeutic experience.” (P6)

“**Did you experience any emotions during the experience?** Excitement, fun and joy […].” (P10)

“[I felt] joy and interest [excitement] or something like that. I don't really know how to describe it.” (P11)

“In the beginning it was very nice.” (P7)

“At first, what I experienced in there, it was interesting and fun. And all of these movements have a [roller coaster] effect, where you’re going up or down at high speeds.” (P13)

“I took pleasure in it. I just enjoyed the experience – so cool. Like a vacation from physical reality, where we have to cooperate with the laws of physics. You didn’t need to do that in there and it was relaxing and enjoyable. Perhaps that also counts as an emotion. [I felt] pleasurable emotions until wanting to fall asleep. It feels enjoyable.” (P13)

“[I felt] nice. Or actually completely numb.” (P5)

Physical tension - 3 (P3, P8, P13)

“[…] If there was simultaneously very intense music and visual – [this caused] a very strong feeling. I understood that it’s smarter or easier to let go at those moments. Without trying to think about where you are or what you are doing, just going with the movement […]” (P3)

“That’s when I felt this intense feeling, as if it was pulling me into it somehow, and also [I felt] a little dizzy. But maybe it was just the first time. Afterwards, I got used to it and I didn’t get that feeling anymore.” (P8)

“At first, what I experienced in there, it was interesting and fun. And all of these movements have a [roller coaster] effect, where you’re going up or down with high speeds. All of this was very intense. All of this [sensations of movement during the experience] was very intense. Almost so intense that you want to hold your arms out in front of yourself or being afraid of falling against something. It was almost so strong that I would’ve liked to scream a little bit. This urge to scream faded away. [I] developed trust that it [the experience] is actually safe.” (P13)

“This visual and auditory intensity of going down some pipe or into some funnel. It was very intense for a moment. The first time was extremely intense, but it helped to focus more on the feeling, not on what you’re seeing.” (P3)

Emotional tension - 4 (P3, P7, P5, P8)

“At one moment I got very emotional. It was during the calmer visual parts.” (P3)

I felt dread related to that one image [the mountains]. Hopelessness. But I think it’s because we had talked about that [personal traumatic] experience then I haven’t really- when I came here yesterday, then I would have said that I don’t feel any- I wasn’t in touch with them [emotions]. Rather, I got in touch with certain negative emotions. (P7)

For a long time I had this scepticism that oh, it’s some experiment and it won’t have an effect on me. [...] I think maybe even half of the time I was holding on or thinking that it is just trying to manipulate my brain. I had a very strong sceptical feeling. […] I had asked this question about my sadness. And then at some point I felt very strong sadness. Or I went into this place of very strong sadness and that’s actually when that whole experience started to work for me. (P12)

[...] Maybe that sadness is what I keep trying to fill with my crazy schedule and many activities. That if you’re scared to get in touch with it or if I can’t bring myself to look at that emotion then that’s when all these things, different things keep coming. I think it might have something to do with that. (P12)

“[...] Well, the music made me sad. I really love the violin and I thought, okay, here come the tears [...]. Then I remembered how I listen to music and that I should do it more, that emotion was very strong.” (P6)

“At one point I had a hysterical desire to laugh. Something made me want to laugh so hard. And then at one point I felt really strong sadness, that was when there were these lines [Mystical Light level]. There was this one bright star and then there were lines everywhere, that made me incredibly sad.” (P5)

“Just before, what could have brought this feeling of fear was that when I was looking at the clouds that had different strange things happening to them, the colours were changing and so on, then I had this apocalyptic feeling or like the world was ending, that is this what the end of the world could be like. And related to that I felt- maybe that’s where the feelings of fear began, this loneliness and fear. [And I was thinking] that maybe this solitude is the end of the world for me.” (P8)

Ambiguous tension - 5 (P4, P10, P1, P8, P9)

“I guess people can be different, or well they definitely are different, but this fast movement of patterns, for me that was very uncomfortable for awhile.” (P4)

“In the beginning I had a feeling of being drunk or when you go to sleep while drunk. Once your head hits the pillow and you close your eyes then that’s the exact same feeling - a carousel. I don’t know if that’s an altered state of consciousness, I guess it is. That was in the beginning. But then at one point I got used to that visual and the movement and then I no longer got that feeling.” (P10)

“[Compared to] everything else that is related to visual art or on a screen, I’ve never experienced them from so close, so it was a very strong experience. I’ve been to panoramic cinemas and all kinds of things in England and all over Europe – like skywatching and all kinds of things. But it’s [the accompanying feeling] has never been so close and so-so strong.” (P6)

For a moment there was this anxiety that I suddenly remembered that I have often dreamt that I’m basically like falling or something, there were several scenes where it’s as if you’re in free fall.” (P1)

“And then, the most fun part is that I experienced pregnancy. You ask if I felt something sensational, I sure did! I managed this big thing. And everything was fine.” (P6)

“One emotion that I can recall which was quite strong - for a moment I even thought that maybe I should remove the [VR] goggles - was this intensity or also a bit of fear when there was this visually very big change from that first [environment] and where the visual shapes started to “run”. That’s when I felt this intense feeling, as if it was pulling me into it somehow, and also [I felt] a little dizzy.” (P8)

“This was at the very beginning where there was a very colourful fractal, that made me feel a little epileptic. I don’t have epilepsy, but my sister for instance does, I’m not sure maybe she would be especially bothered by that.” (P9)

SM5. Measures

To evaluate the depressive symptoms of the subjects we used the updated version of The Emotional State Questionnaire (EST-Q2; originally reported in[3]) with 28 self-report items assessing dimensions of depression, general anxiety, agoraphobia–panic, fatigue, and insomnia. The items are derived from the diagnostic criteria presented in the Diagnostic and Statistical Manual of Mental Disorders, 4th Edition (DSMIV) and the International Classification of Diseases, 10th Edition (ICD-10), and the subscales have demonstrated overall good reliability and discriminative validity when comparing patients with mood and anxiety disorders with a population sample. EST-Q2 is a well-established questionnaire to measure symptoms of depression and anxiety in research (e.g., [4,5]) and is also the most frequently used self-administered screening tool for depression among Estonian healthcare professionals (Puis et al., 2017). The EST-Q2 was used to recruit subjects who reported mild-to-moderate depressive symptoms from the previous month (using the depressive scale cut-off of ≥ 12[5]), as well as to test any change in symptoms two weeks after the experiments. The Estonian translation of the Montgomery-Åsberg Depression Scale[6] (MADRS) was also administered during the first day by the psychologist to validate the severity of depression of the subject with an alternative measure.

The Revised Mystical Experience Questionnaire[7–9] (MEQ30) was used to measure the intensity of mystical experience. It has been used with psychedelics and is well-validated for use in the context of psychedelic experiences with high scores predicting positive persisting change in attitudes, behaviour and well-being[10]. MEQ30 has 4 subscales: Mystical, Ineffability, Transcendence of Space and Time and Positive Mood. It includes questions such as "Experience of pure being and pure awareness (beyond the world of sense impressions)" (Item 16), "Experience of unity with ultimate reality" (Item 28) and "Sense of being "outside of" time, beyond past and future" (Item 13).

The Psychological Insight Questionnaire[11] (PIQ) is a recently developed questionnaire that is created specifically to investigate acute insight experiences that occur during psychedelic experiences. It consists of two subscales: Avoidance and Maladaptive Patterns Insights (AMP) and Goals and Adaptive Patterns Insights (GAP). First studies have shown that the PIQ average score and AMP subscale scores predict changes in psychological flexibility and PIQ, AMP and GAP scores predict changes in well-being and life satisfaction. PIQ includes questions such as "Awareness of uncomfortable or painful feelings I previously avoided" (AMP), "Discovered clarity or creative solutions about how to solve a problem in my life" (GAP) and "Gained resolution or clarity about past traumas or hurtful events" (AMP).
To estimate whether participants experienced ego dissolution during the experiment we used the Ego Dissolution Inventory[12] (EDI). EDI is an 8-item scale that is designed to capture the ego-dissolution experience based on research on psychedelics. It includes statements that relate to the ego dissolution experience such as "I experienced a dissolution of my "self" or ego", "I felt at one with the universe" and "I felt far less absorbed by my own issues and concerns."

SM6. Study setting

The experimental design was strongly influenced by previous research into similar methods[13,14] and current ideas regarding psychedelic-assisted therapy emphasizing the effect of “set and setting“[15–23]. “Set” refers to the participants' state of mind before and during the experience, being influenced by preparations, mood and personality traits[16,19]. “Setting” covers the physical, social and cultural environment where the experience takes place[16,19]. Virtual reality, however, allows to somewhat relieve the preparational demands on the physical environment, as the focal part of the setting is virtual. So the laboratory room was largely left unchanged from its usual professional and sterile decor, but some plants were added and a comfortable suspended chair was used for seating during the experience (Fig S8). The first day was to help the participants get used to the environment and to build rapport and trust with the researchers to minimize any unnecessary discomfort or anxiety and to allow the participants to feel safe and relaxed[20,24]. Furthermore, akin to contemporary approaches in psychedelic therapy[24–26], the participants were prepared for any unpleasant experiences (cybersickness or emotional reactions) beforehand. They were encouraged to direct their attention towards any feelings that might arise and “let those feelings go“[20,25,26]. A short twelve minute guided meditation was conducted prior to the experience to induce openness and calmness in the participants as well as to enhance their immersion[27–30].

A clinical psychologist was present during the experiments for conducting an assessment of depressive symptoms, aiding with the evaluation of therapeutic effects and in case of psychological emergencies. The psychologist avoided using any therapeutic techniques (validation, reflection, etc) and assumed the role of an observer and guide[24,31]. Importantly, the psychologist also helped the participants to define questions related to their symptoms to see whether such methods can help people arrive at answers for specific pathology-related questions.

Integration of the experience is considered to be a vital part of psychedelic-assisted therapies[31–33]. Based on this we also implemented an integrative discussion in the form of a semi-structured interview after the VR experience, where the participant was encouraged to freely discuss their experience along with some guiding questions regarding emotions, thoughts or alterations in the sense of self and consciousness. The integrative session was recorded with permission from the participant and later transcribed. A thematic analysis was conducted on the transcriptions from the integration session. In case of a psychological emergency, the psychologist was also prepared to offer further support and guidance.

SM7. Overview of Psyrreal VR

A custom soundtrack, produced by an Estonian composer specifically for Psyrreal, musically depicted different elements from psychedelic phenomenology. Among others, this includes smooth and/or sudden transitions, looping/recurrence/fractal effects and varying intensity[34]. The Shepard-Risset glissando illusion[35,36] (Risset, 1969; Shepard, 1964) was used in multiple places to imitate the auditory phenomenology of the 'DMT breakthrough' (see "DMT hyperspace", table 1) and induce sensations of intensity[37]. Varying the intensity and tempo of the soundtrack was also employed to create a sense of temporal alteration. Additionally, music that has been previously used for psychedelic therapy served as a source of inspiration, especially in constructing calmer parts of the experience. The visual levels were built to match the composed soundtrack, emphasizing audiovisual synchronization to imitate the synesthetic elements common in psychedelic experiences[38–40].

The narrative sequence was based on the progression of psychedelic (mainly LSD and psilocybin) experiences which have a longer duration and a smoother onset, but elements from DMT experiences were also implemented (e.g., the "DMT breakthrough", see table 1). The experience starts in a virtual "real world" ("Main" in Fig 1) which starts to acquire psychedelic phenomenology in an oscillating amplification[34]. As the effects become more intense, at around 7 minutes the environment changes into an imitation of being with eyes closed, with the appearance of closed-eye visuals of colorful 2D patterns which slowly amplify in vividness and intensity, further evolving into 3D patterned surroundings. The experience is for the most part abstract, avoiding elements with direct meaning. We have designed the experience using mostly conceptual shapes and patterns that would allow participants to project their own meaning to the experience. This is similar to what often happens as the psychedelic experience intensifies, with geometric patterns forming objects or sceneries and developing contextual meaning related to the prior memories and experiences of the participant[41,42]. As the experience progresses, levels with dreamlike visionary images[43,44] and more meaningful grandiose environments[41,42,45] follow. Some shorter, visually simpler levels with calm and sacred music were also included, which imitate the less complex audiovisual phenomenology of mystical experiences. The end of the experience was based on the comedown of psychedelic experiences, returning to the initial main "real" virtual environment, with the amplitude of visual effects slowly decaying.

References

1. SteamVR Home Beta now available. https://steamcommunity.com/games/250820/announcements/detail/1319965970589887588 (2017).

2. The Lab, developed by Valve Corporation.

3. Aluoja, A., Shlik, J., Vasar, V., Luuk, K. & Leinsalu, M. Development and psychometric properties of the Emotional State Questionnaire, a self-report questionnaire for depression and anxiety. *Nord. J. Psychiatry* (1999) doi:10.1080/080394899427692.

4. Eller, T., Aluoja, A., Vasar, V. & Veldi, M. Symptoms of anxiety and depression in Estonian medical students with sleep problems. *Depress. Anxiety* (2006) doi:10.1002/da.20166.

5. Ööpik, P., Aluoja, A., Kalda, R. & Maaroos, H. I. Screening for depression in primary care. *Fam. Pract.* (2006) doi:10.1093/fampra/cml052.

6. Davidson, J., Turnbull, C. D., Strickland, R., Miller, R. & Graves, K. The Montgomery-Åsberg Depression Scale: reliability and validity. *Acta Psychiatr. Scand.* (1986) doi:10.1111/j.1600-0447.1986.tb02723.x.

7. Griffiths, R. R., Richards, W. A., McCann, U. & Jesse, R. Psilocybin can occasion mystical-type experiences having substantial and sustained personal meaning and spiritual significance. *Psychopharmacology (Berl).* (2006) doi:10.1007/s00213-006-0457-5.

8. Landes, H. Õnnelikkuse seosed religioossuse ja müstilise kogemusega Eesti valimil. (University of Tartu, 2020).

9. MacLean, K. A., Leoutsakos, J. M. S., Johnson, M. W. & Griffiths, R. R. Factor Analysis of the Mystical Experience Questionnaire: A Study of Experiences Occasioned by the Hallucinogen Psilocybin. *J. Sci. Study Relig.* (2012) doi:10.1111/j.1468-5906.2012.01685.x.

10. Barrett, F. S., Johnson, M. W. & Griffiths, R. R. Validation of the revised Mystical Experience Questionnaire in experimental sessions with psilocybin. *J. Psychopharmacol.* (2015) doi:10.1177/0269881115609019.

11. Davis, A. K. *et al.* Development of the Psychological Insight Questionnaire among a sample of people who have consumed psilocybin or LSD. *J. Psychopharmacol.* (2021) doi:10.1177/0269881120967878.

12. Nour, M. M., Evans, L., Nutt, D. & Carhart-Harris, R. L. Ego-dissolution and psychedelics: Validation of the ego-dissolution inventory (EDI). *Front. Hum. Neurosci.* (2016) doi:10.3389/fnhum.2016.00269.

13. Carhart-Harris, R. L. *et al.* Psilocybin with psychological support for treatment-resistant depression: an open-label feasibility study. *The Lancet Psychiatry* (2016) doi:10.1016/S2215-0366(16)30065-7.

14. Falconer, C. J. *et al.* Embodying self-compassion within virtual reality and its effects

on patients with depression. *BJPsych Open* (2016) doi:10.1192/bjpo.bp.115.002147.

15. Carhart-Harris, R. L. *et al.* LSD enhances suggestibility in healthy volunteers. *Psychopharmacology (Berl).* (2015) doi:10.1007/s00213-014-3714-z.

16. Carhart-Harris, R. L. *et al.* Psychedelics and the essential importance of context. *J. Psychopharmacol.* (2018) doi:10.1177/0269881118754710.

17. Gukasyan, N. & Nayak, S. M. Psychedelics, placebo effects, and set and setting: Insights from common factors theory of psychotherapy. *Transcultural Psychiatry* (2021) doi:10.1177/1363461520983684.

18. Hartogsohn, I. Set and setting, psychedelics and the placebo response: An extra-pharmacological perspective on psychopharmacology. *Journal of Psychopharmacology* (2016) doi:10.1177/0269881116677852.

19. Hartogsohn, I. Constructing drug effects: A history of set and setting. *Drug Sci. Policy Law* (2017) doi:10.1177/2050324516683325.

20. Johnson, M. W., Richards, W. A. & Griffiths, R. R. Human hallucinogen research: Guidelines for safety. *Journal of Psychopharmacology* (2008) doi:10.1177/0269881108093587.

21. Kaelen, M. *et al.* The hidden therapist: evidence for a central role of music in psychedelic therapy. *Psychopharmacology (Berl).* (2018) doi:10.1007/s00213-017-4820-5.

22. Leary, T., Litwin, G. H. & Metzner, R. Reactions to psilocybin administered in a supportive environment. *J. Nerv. Ment. Dis.* (1963) doi:10.1097/00005053-196312000-00007.

23. Uthaug, M. V. *et al.* A placebo-controlled study of the effects of ayahuasca, set and setting on mental health of participants in ayahuasca group retreats. *Psychopharmacology (Berl).* (2021) doi:10.1007/s00213-021-05817-8.

24. Garcia-Romeu, A. & Richards, W. A. Current perspectives on psychedelic therapy: use of serotonergic hallucinogens in clinical interventions. *International Review of Psychiatry* (2018) doi:10.1080/09540261.2018.1486289.

25. Watts, R., Day, C., Krzanowski, J., Nutt, D. & Carhart-Harris, R. Patients' Accounts of Increased "Connectedness" and "Acceptance" After Psilocybin for Treatment-Resistant Depression. *J. Humanist. Psychol.* (2017) doi:10.1177/0022167817709585.

26. Wolff, M. *et al.* Learning to Let Go: A Cognitive-Behavioral Model of How Psychedelic Therapy Promotes Acceptance. *Front. Psychiatry* (2020) doi:10.3389/fpsyt.2020.00005.

27. Döllinger, N., Wienrich, C. & Latoschik, M. E. Challenges and Opportunities of Immersive Technologies for Mindfulness Meditation: A Systematic Review. *Front. Virtual Real.* (2021) doi:10.3389/frvir.2021.644683.

28. Heeter, C. & Allbritton, M. Being There: Implications of Neuroscience and Meditation for Self-Presence in Virtual Worlds. *J. Virtual Worlds Res.* (2015) doi:10.4101/jvwr.v8i2.7164.

29. Imtiaz, F., Ji, L. J. & Vaughan-Johnston, T. Exploring the influence of a low-dose mindfulness induction on performance and persistence in a challenging cognitive task. *J. Theor. Soc. Psychol.* (2018) doi:10.1002/jts5.32.

30. Miller, M., Mistry, D., Jetly, R. & Frewen, P. Meditating in Virtual Reality 2: Phenomenology of Vividness, Egocentricity and Absorption-Immersion. *Mindfulness (N. Y).* (2021) doi:10.1007/s12671-020-01589-y.

31. Gorman, I., Nielson, E. M., Molinar, A., Cassidy, K. & Sabbagh, J. Psychedelic Harm Reduction and Integration: A Transtheoretical Model for Clinical Practice. *Front. Psychol.* (2021) doi:10.3389/fpsyg.2021.645246.

32. Belser, A. B. *et al.* Patient Experiences of Psilocybin-Assisted Psychotherapy: An Interpretative Phenomenological Analysis. *J. Humanist. Psychol.* (2017) doi:10.1177/0022167817706884.

33. Grof, S. *LSD Psychotherapy*. (Hunter House, 1980).

34. Kins, J. Subjective Effect Index. https://effectindex.com/.

35. Risset, J. C. Pitch Control and Pitch Paradoxes Demonstrated with Computer-Synthesized Sounds. *J. Acoust. Soc. Am.* (1969) doi:10.1121/1.1973626.

36. Shepard, R. N. Circularity in Judgments of Relative Pitch. *J. Acoust. Soc. Am.* (1964) doi:10.1121/1.1919362.

37. Mursic, R. A. & Palmisano, S. The Shepard-Risset Glissando: Identifying the Origins of Metaphorical Auditory Vection and Motion Sickness. *Multisens. Res.* (2020) doi:10.1163/22134808-20191450.

38. Brogaard, B. & Gatzia, D. E. Psilocybin, Lysergic Acid Diethylamide, Mescaline, and Drug-Induced Synesthesia. in *Neuropathology of Drug Addictions and Substance Misuse* (2016). doi:10.1016/B978-0-12-800212-4.00083-2.

39. Sinke, C. *et al.* Genuine and drug-induced synesthesia: A comparison. *Consciousness and Cognition* (2012) doi:10.1016/j.concog.2012.03.009.

40. Terhune, D. B. *et al.* A placebo-controlled investigation of synaesthesia-like experiences under LSD. *Neuropsychologia* (2016) doi:10.1016/j.neuropsychologia.2016.04.005.

41. Preller, K. H. & Vollenweider, F. X. Phenomenology, structure, and dynamic of psychedelic states. in *Current Topics in Behavioral Neurosciences* (2018). doi:10.1007/7854_2016_459.

42. Masters, R. E. L. & Houston, J. *The Varietes of psychedelic experience*. (Dell Publishing, 1966).

43. Kraehenmann, R. *et al.* Dreamlike effects of LSD on waking imagery in humans depend on serotonin 2A receptor activation. *Psychopharmacology (Berl).* (2017) doi:10.1007/s00213-017-4610-0.

44. Sanz, C. & Tagliazucchi, E. The experience elicited by hallucinogens presents the highest similarity to dreaming within a large database of psychoactive substance reports. *Front. Neurosci.* (2018) doi:10.3389/fnins.2018.00007.

45. Keltner, D. & Haidt, J. Approaching awe, a moral, spiritual, and aesthetic emotion. *Cognition and Emotion* (2003) doi:10.1080/02699930302297.
